# Characterization of adaptive evolution strains for the development of triclosan resistance in *Agrobacterium tumefaciens* C58

**DOI:** 10.1128/aem.01232-25

**Published:** 2026-01-06

**Authors:** Nathapol Tasnawijitwong, Benya Nontaleerak, Kwanrawee Sirikanchana, Jutamaad Satayavivad, Rojana Sukchawalit, Skorn Mongkolsuk

**Affiliations:** 1 Program in Environmental Toxicology, Chulabhorn Graduate Institute 251502, Bangkok, Thailand; 2 Laboratory of Biotechnology, Chulabhorn Research Institute 67969, Bangkok, Thailand; 3 Center of Excellence on Environmental Health and Toxicology (EHT), OPS, MHESI, Bangkok, Thailand; 4 Laboratory of Pharmacology, Chulabhorn Research Institute 67969, Bangkok, Thailand; 5 Program in Applied Biological Sciences, Chulabhorn Graduate Institute 251502, Bangkok, Thailand; Washington University in St. Louis, St. Louis, Missouri, USA

**Keywords:** adaptive triclosan resistance, TriABC efflux pump, biofilm, antibiotic resistance

## Abstract

*Agrobacterium tumefaciens,* a soil bacterium, was used as a model organism to study the mechanisms of triclosan (TCS) resistance in environmental bacteria. Adaptive laboratory evolution tests were performed to select TCS-resistant strains by challenging a wild-type (WT) strain with increasing concentrations of TCS (8, 12, 16, and 20 µg/mL). Two high-dose-resistant strains, HDR-12a and HDR-20a, were isolated and used for detailed examination. In comparison to the minimum inhibitory concentration of the WT strain (10 µg/mL), HDR-12a (20 µg/mL), and HDR-20a (32 µg/mL) showed increased resistance to TCS. Whole-genome sequencing and transcriptomic analysis performed to identify mechanisms underlying the different degrees of TCS resistance among the two evolved *A. tumefaciens* strains revealed a nucleotide base change (missense mutation, Asn157Thr) in the transcriptional repressor *triR* gene as the key mechanism of TCS resistance in HDR-20a. This change reduced the DNA-binding ability of TriR, causing overexpression of the *triABC* operon that encodes the TCS-specific efflux pump. In contrast, HDR-12a had no mutation in the *triR* gene. HDR-12a exhibited transcriptomic changes in several genes involved in ATP-binding cassette (ABC) transporters and in the metabolism of sulfur, fatty acids, and carbohydrates. However, it remains unclear whether these transcriptomic changes are directly responsible for TCS resistance in HDR-12a. Both the TCS-adapted strains also showed increased resistance to chloramphenicol and erythromycin. Overall, these results demonstrate that TCS pollution in environmental hotspots can select for adaptive and cross-resistant bacteria.

**IMPORTANCE:**

TCS is widely used as a preservative and disinfectant in many personal healthcare products. TCS is subsequently released into aquatic and terrestrial environments. The emergence and spread of multidrug-resistant pathogens from the use of antimicrobials like TCS and the misuse of antibiotic drugs now pose a serious global public health threat. Understanding how resistance develops has implications for preventing the emergence of antimicrobial resistance. The adapted TCS-resistant strains showed cross-resistance to chloramphenicol and erythromycin. This study provides insight into how environmental exposure to triclosan can drive adaptive and cross-resistance mechanisms in a soil bacterium, highlighting its relevance to environmental antimicrobial resistance and public health risk.

## INTRODUCTION

Triclosan (TCS), or 5-chloro-2-(2,4-dichlorophenoxy) phenol, is a synthetic bisphenolic biocide that is widely used as an effective broad-spectrum antibacterial agent (1). TCS is added to personal healthcare products, household cleaning products, medical devices, and clinical supplies; however, this extensive use has resulted in massive releases of TCS into aquatic and terrestrial environments (2, 3). The released TCS also bioaccumulates and persists in the environment, with adverse effects on human health (4, 5). Furthermore, increasing evidence now indicates that TCS exposure selects for TCS-resistant bacteria while also promoting antibiotic resistance (4). The emergence and spread of multidrug-resistant pathogens from the use of antimicrobials like TCS and the misuse of antibiotic drugs now pose a serious global public health threat (6).

TCS rapidly permeates bacterial cell walls and disrupts multiple cellular processes. Its bacteriostatic action at low concentrations is attained by specific inhibition of the enoyl-acyl carrier protein reductase (ENR) enzyme involved in fatty acid synthesis and lipid metabolism (7, 8). At higher concentrations (>10 µg/mL), TCS becomes bactericidal by intercalating into the bacterial cell membranes and disrupting membrane integrity (9, 10). Microbial resistance to these actions of TCS can arise through intrinsic or acquired mechanisms (11). For example, in gram-negative bacteria, the outer membrane acts as a permeability barrier that prevents the entry of toxic compounds while allowing the influx of needed nutrient molecules (12, 13).

Several adaptive mechanisms for TCS resistance, such as target gene mutation, overexpression of *fabI*, induction of efflux pumps, TCS transformation and degradation, decreased membrane permeability, and biofilm formation, have been identified in bacteria (14). Multidrug resistance efflux pumps can excrete different classes of drugs and numerous structurally unrelated substrates (15). In gram-negative bacteria, the multidrug resistance efflux pump is a complex consisting of a periplasmic membrane fusion protein (MFP), an inner membrane transporter, and an outer membrane factor (OMF) protein, enabling antibiotic extrusion across the double-membrane cell envelope. The TriABC-OpmH complex, first reported in *Pseudomonas aeruginosa*, is a TCS-specific efflux system that requires two MFPs (TriA and TriB) and an outer membrane channel (OpmH) to function (16). TriA is involved in the recruitment of OpmH and in stabilizing interactions with OpmH, whereas TriB is required for the stimulation of TriC, a resistance–nodulation–division (RND)-type efflux transporter (17, 18). TriABC has been demonstrated in *P. aeruginosa*, but its regulatory molecular mechanism has not been reported. *P. aeruginosa* can also extrude TCS through several other multidrug resistance efflux pump systems, including MexAB-OprM, MexCD-OprJ, MexEF-OprN, and MexJK-OmpH (19, 20). In other bacteria, TCS is extruded by several efflux pumps, including CmeABC in *Campylobacter jejuni* (21), SmeDEF in *Stenotrophomonas maltophilia* (22), BmeABC in *Bacteroides fragilis* (23), OqxAB in *Klebsiella pneumoniae* (24), and AdeB in *Acinetobacter baumannii* (25).

*Agrobacterium tumefaciens* C58, also known as *A. fabrum*, is a soil bacterium that causes crown gall disease in plants, and it senses TCS specifically through the transcriptional regulator TriR (26), which represses the *triABC* operon that encodes the TCS-specific efflux pump. Upon binding to TCS, TriR loses its ability to bind DNA, leading to the induction of the *triABC* gene in response to TCS exposure (26). The resulting overexpression of TriABC confers a high level of resistance to TCS while imparting cross-resistance to the detergent sodium dodecyl sulfate (SDS) and the quaternary ammonium compound benzalkonium chloride (26). In *A. tumefaciens*, TCS can also be sensed by AcrR, the transcriptional repressor of genes coding for the AcrAB efflux pump (27). However, the disruption of AcrAB slightly increases the sensitivity of *A. tumefaciens* to TCS, suggesting that TriABC is the major TCS efflux pump in this bacterium. Furthermore, the response to TCS was weaker for *A. tumefaciens* AcrR than for TriR (27).

During the COVID-19 pandemic, massive amounts of used disinfectants, including TCS, were released into sewers and entered wastewater treatment plants (WWTPs). TCS eventually entered the environment mainly through WWTP effluent release into aquatic systems and the deposition of biosolids in soil as agricultural fertilizers (28). WWTP biosolids are also enriched in chemical pollutants (e.g., biocides and heavy metals), antibiotics, and microbes carrying mobile genetic elements and associated resistance genes (29). Therefore, biosolids are regarded as hot spots of horizontal gene transfer between clinical and environmental bacteria and may pose risks of direct gene transfer to humans (30). TCS pollution could increase the selective pressure for microbial resistance to antibiotics and potentially have negative health and environmental effects (14, 28). Overall, understanding the mechanisms of bacterial adaptation to TCS exposure and elucidating the potential for cross-resistance to biocides and antibiotics could help prevent the emergence and spread of multidrug-resistant pathogens in response to TCS pollution.

One potentially useful approach for revealing the evolution of stress-tolerance mechanisms in bacteria is adaptive laboratory evolution (31) performed by sequential bacterial passages with increasing stressor concentrations to enable selection of cells most adapted to the stressor. Previous studies have used antibiotics at sub-minimum inhibitory concentrations (MICs) for rapid development of antimicrobial resistance (31–34). Here, our aim was to exploit adaptive laboratory evolution to enable the identification of mechanisms by which *A. tumefaciens* develops TCS resistance.

In this study, TCS-resistant strains were isolated through adaptive laboratory evolution by initially treating *A. tumefaciens* with TCS at a sub-MIC concentration (8 µg/mL), and then gradually increasing the TCS concentration to 12, 16, and 20 µg/mL. The underlying mechanisms leading to different degrees of TCS resistance were revealed by whole genome sequencing and transcriptome analysis of the evolved strains.

## RESULTS

### Selection of adaptive TCS-resistant strains HDR-12a and HDR-20a

**Fig 1:**
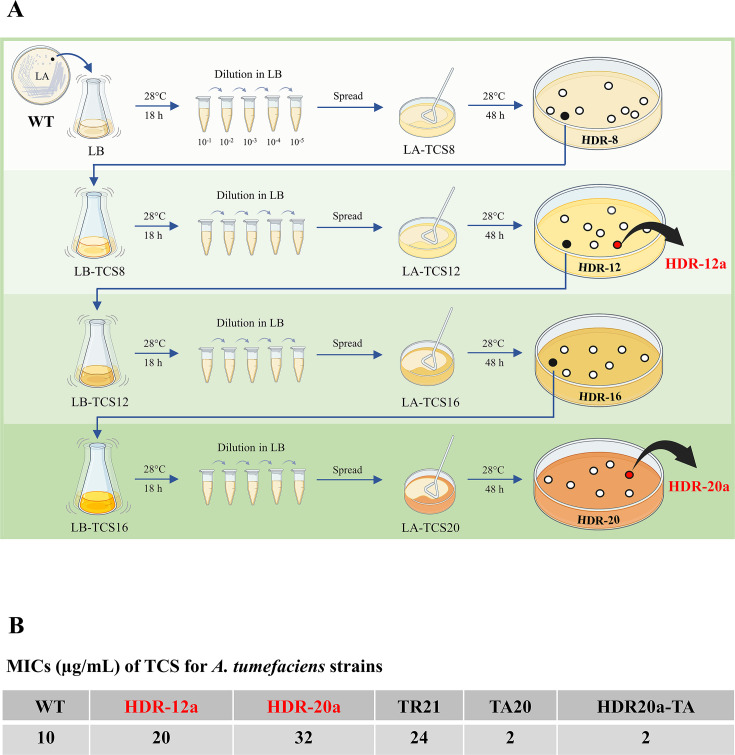
Adaptive triclosan (TCS)-resistant strains HDR-12a and HDR-20a. (**A**) Schematic representation of adaptive laboratory evolution for isolating TCS-resistant strains. The adaptive resistance strains were isolated by exposure to gradually increasing concentrations of TCS (8, 12, 16, and 20 µg/mL). First, a single colony of the wild-type (WT) NTL4 strain was cultured in LB medium with shaking at 28°C for 18 h. The cell culture was then serially diluted tenfold in fresh LB, and 100 µL of a 1 × 10^−5^ dilution was spread on an LB agar (LA) plate containing 8 µg/mL of TCS (LA-TCS8). After incubation at 28°C for 48 h, the surviving colonies were defined as high-dose resistant-8 (HDR-8) cells. A single colony from the LA-TCS8 plate was picked and cultured in fresh LB broth containing 8 µg/mL TCS (LB-TCS8) with shaking at 28°C for 18 h. The resulting cell culture was then diluted in fresh LB and spread on an LA plate containing 12 µg/mL of TCS (LA-TCS12) to select HDR-12 cells. The HDR-16 and HDR-20 cells were similarly generated by subsequent selections. The two adaptive TCS-resistant strains named HDR-12a and HDR-20a were single colonies picked from the LA-TCS12 and LA-TCS20 plates, respectively. (**B**) Determination of the minimum inhibitory concentration (MIC) using the agar dilution method. Exponential growth phase cells of WT, HDR-12a, HDR-20a, TR21 (*triR* disruption), TA20 (*triA* disruption), and HDR20a-TA (HDR-20a with *triA* disruption) were adjusted, serially diluted tenfold, and grown on LB agar (LA) plates containing TCS (1–32 μg/mL in increments of 2) and incubated at 28°C for 48 h (Fig. S2). The MIC was defined as the lowest concentration of TCS that inhibited the visible growth of bacteria. The experiment was repeated twice.

The mechanisms involving TCS resistance in *A. tumefaciens* were investigated by isolating high-dose–resistant (HDR) strains of *A. tumefaciens*. The selection procedure is described in Fig. 1A. The MIC for TCS, determined using the agar dilution method, was 10 µg/mL for the WT NTL4 strain (Fig. S1). TCS at the sub-MIC concentration of 8 µg/mL was used as the initial concentration for the selection process. Determination of the TCS MIC for the *A. tumefaciens* strains (WT, HDR-12a, HDR-20a, TR21, TA20, and HDR20a-TA) (Fig. S1) confirmed that both HDR-12a (20 µg/mL) and HDR-20a (32 µg/mL) cells exhibited increased TCS resistance (increased MIC values) when compared with the WT cells (10 µg/mL) (Fig. 1B). The TR21 (*triR* disruption, MIC 24 µg/mL) and TA20 (*triA* disruption, MIC 2 µg/mL) strains from a previous study (26) were used as controls.

### Mutations in the HDR-12a and HDR-20a genomic DNA

**Fig 2:**
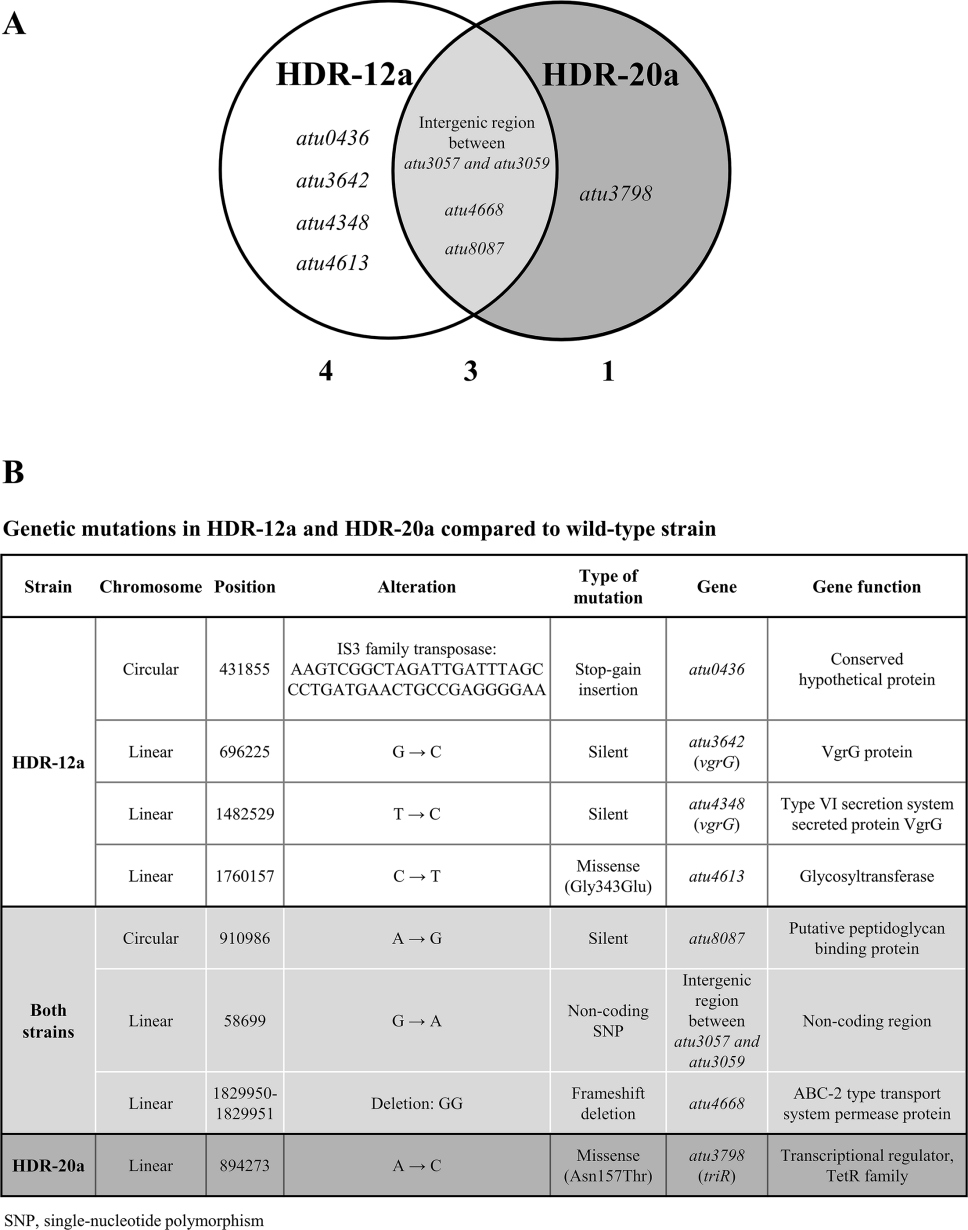
Whole-genome sequencing (WGS) analysis. Genetic mutations occurring during TCS adaptation were identified by comparing the genome sequences of HDR-12a and HDR-20a cells with the genome sequence of the WT cells. (**A**) A Venn diagram showing the number of mutations in unique and common genes detected in HDR-12a and HDR-20a cells. The white circle depicts the mutations identified in HDR-12a cells, and the gray circle depicts the mutations identified in HDR-20a cells. The overlapping area indicates shared mutations. (**B**) A list of genetic mutations detected by WGS in HDR-12a and HDR-20a cells.

WGS was performed to identify genetic mutations arising during adaptive laboratory evolution, possibly responsible for the TCS-resistant HDR-12a and HDR-20a phenotypes. Comparison of genomic DNA sequences between the TCS-resistant strains and their parental strain (WT) revealed three mutations in common in HDR-12a and HDR-20a (Fig. 2A): a single-nucleotide polymorphism (SNP) in *atu8087* (putative peptidoglycan binding protein), a non-coding SNP in the intergenic region between *atu3057* and *atu3059*, and a frameshift deletion in *atu4668* (ABC transport system permease protein) (Fig. 2B). Four unique mutations were found in HDR-12a: an IS3 family transposase insertion in *atu0436* (hypothetical protein) and an SNP in each of *atu3642* (VgrG), *atu4348* (VgrG), and *atu4613* (glycosyltransferase) (Fig. 2A and B). The missense mutation (Asn157Thr) in the coding region of *triR* (*atu3798*) was unique to HDR-20a (Fig. 2A and B).

The WGS results implied that both adaptive TCS-resistance strains may have a defective ABC transporter (of unknown function) due to a frameshift deletion in *atu4668*. HDR-12a might have additional functional defects in its glycosyltransferase (*atu4613*, a missense mutation, Gly343Glu) and an unknown protein (*atu0436*, a stop-gain insertion).

### A single base mutation in the *triR* gene of HDR-20a enhanced the expression of the *triABC* efflux pump and conferred high TCS resistance

**Fig 3:**
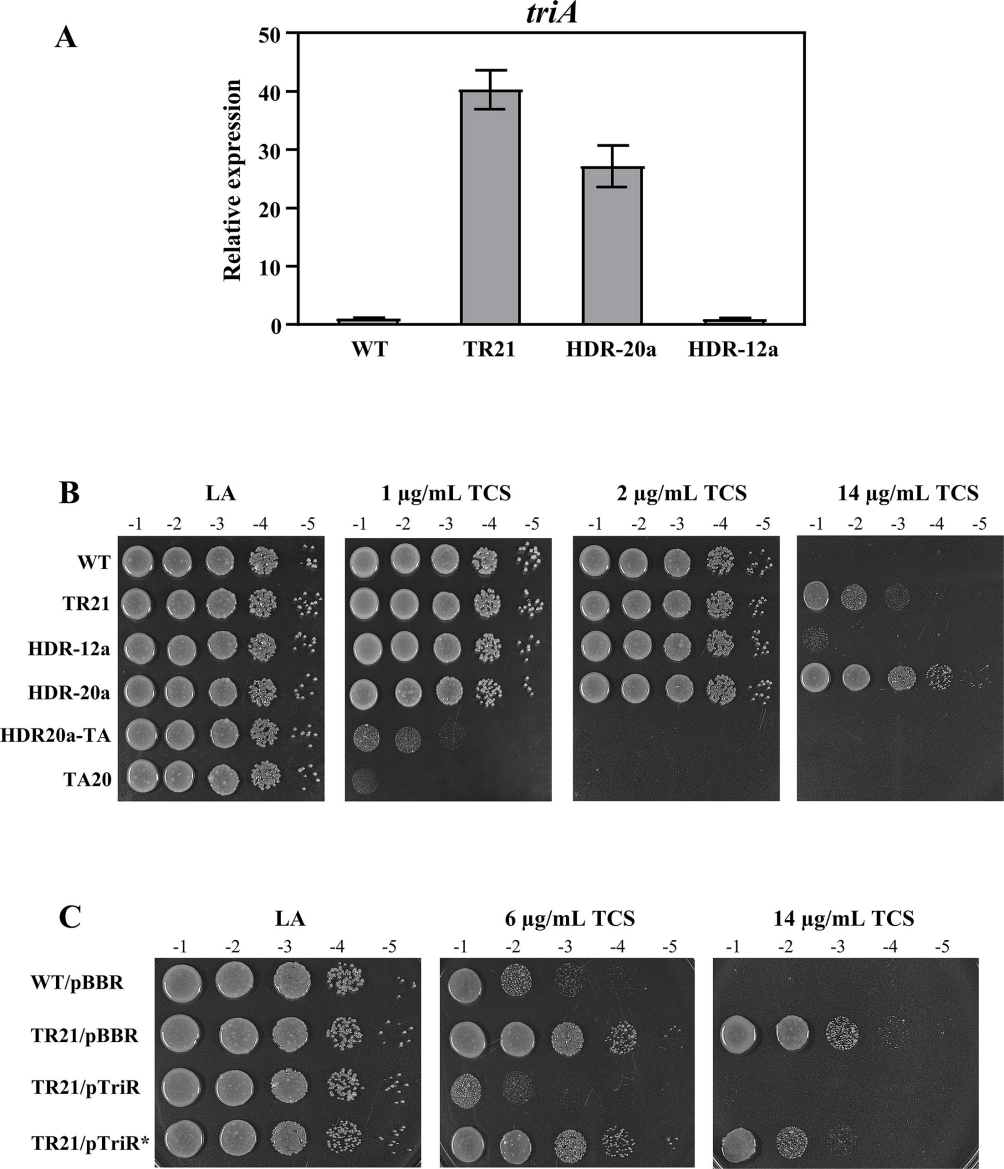
A missense mutation in the *triR* gene led to increased expression of *triABC* and a defect in the TriR function that confers TCS resistance in HDR-20a. (**A**) Quantitative real-time polymerase chain reaction (qRT-PCR). Expression of *triA* levels in exponential-phase cells of WT, TR21 (*triR* disruption), HDR-20a, and HDR-12a cells grown in LB. Fold changes in *triA* expression were relative to expression in the WT cells (regarded as 1). The results represent the means of triplicate independent samples ± SD. (**B** and **C**) Plate sensitivity assays. Exponential-phase cells grown in LB were serially diluted and spotted onto LA plates and LA plates containing TCS at 1, 2, 6, and 14 μg/mL. Tenfold serial dilutions are indicated. The plates were incubated at 28°C for 48 h. Strains were WT, TR21 (*triR* disruption), HDR-12a, HDR-20a, HDR20a-TA (HDR-20a with *triA* disruption), and TA20 (*triA* disruption). Bacterial strains carried either the plasmid vector pBBR, a functional *triR* gene on the pTriR plasmid, or a mutated *triR* gene (triR*, N157T mutation) on the pTriR* plasmid.

The high TCS resistance of HDR-20a was possibly due to the overexpression of the TCS-specific efflux pump *triABC* as a result of *triR* mutation (Asn157Thr). This possibility was tested using qRT-PCR analysis to determine *triA* (the first gene of the *triABC* operon) expression levels using exponential growth phase cells grown in LB medium. In contrast to HDR-12a, HDR-20a exhibited an approximately 27-fold increase in the expression of *triA* compared with the WT strain (Fig. 3A). The previously constructed *triR* mutant (TR21, *triR* disruption) (26) showed approximately 39-fold elevated *triA* expression compared with the WT strain (Fig. 3A). Although HDR-20a had lower *triA* expression than TR21, HDR-20a was more resistant than TR21 (~10^2^-fold) to 14 μg/mL TCS (Fig. 3B).

The TriR homology model was predicted previously on the SWISS-MODEL webserver using the crystal structure of *Stenotrophomonas maltophilia* SmeT (PDB ID: 3P9T) as the template (26). The 3D structure model of TriR consists of nine α helices: an N-terminal DNA binding domain (helices 1–3) and a C-terminal regulatory or ligand-binding domain (helices 4–9). Helices 2 and 3 form a helix-turn-helix motif that interacts with DNA. Helices 5, 6, and 7 form a central triangle, whereas helices 8 and 9 are responsible for dimerization. Asn157 is located at the end of a loop preceding helices 8 and 9 (Fig. S2). The next experiments tested whether the mutated *triR* gene (hereafter designated as triR^*^, a single base change from A to C resulting in amino acid residue 157 change from Asn to Thr) could cause a defect in TriR repressor function, which in turn would contribute to the increased expression of *triABC* in HDR-20a. The function of mutated *triR* was assessed by cloning the triR^*^ gene from HDR-20a into a plasmid expression vector (pBBR) to generate the pTriR* plasmid. Previous work has shown that the *triR* mutant strain (TR21) has higher resistance than the WT strain to TCS (26). Consistent with this previous finding, the resistant phenotype of the *triR* mutant (TR21/pBBR) could be fully reversed by expressing functional *triR* (WT) from the plasmid pTriR (TR21/pTriR) (Fig. 3C). In contrast, complementation with pTriR* (TR21/pTriR*) did not fully reverse the resistant phenotype of the *triR* mutant (Fig. 3C). These results indicated that the mutated *triR* functioned poorly compared with the wild-type *triR*.

**Fig 4:**
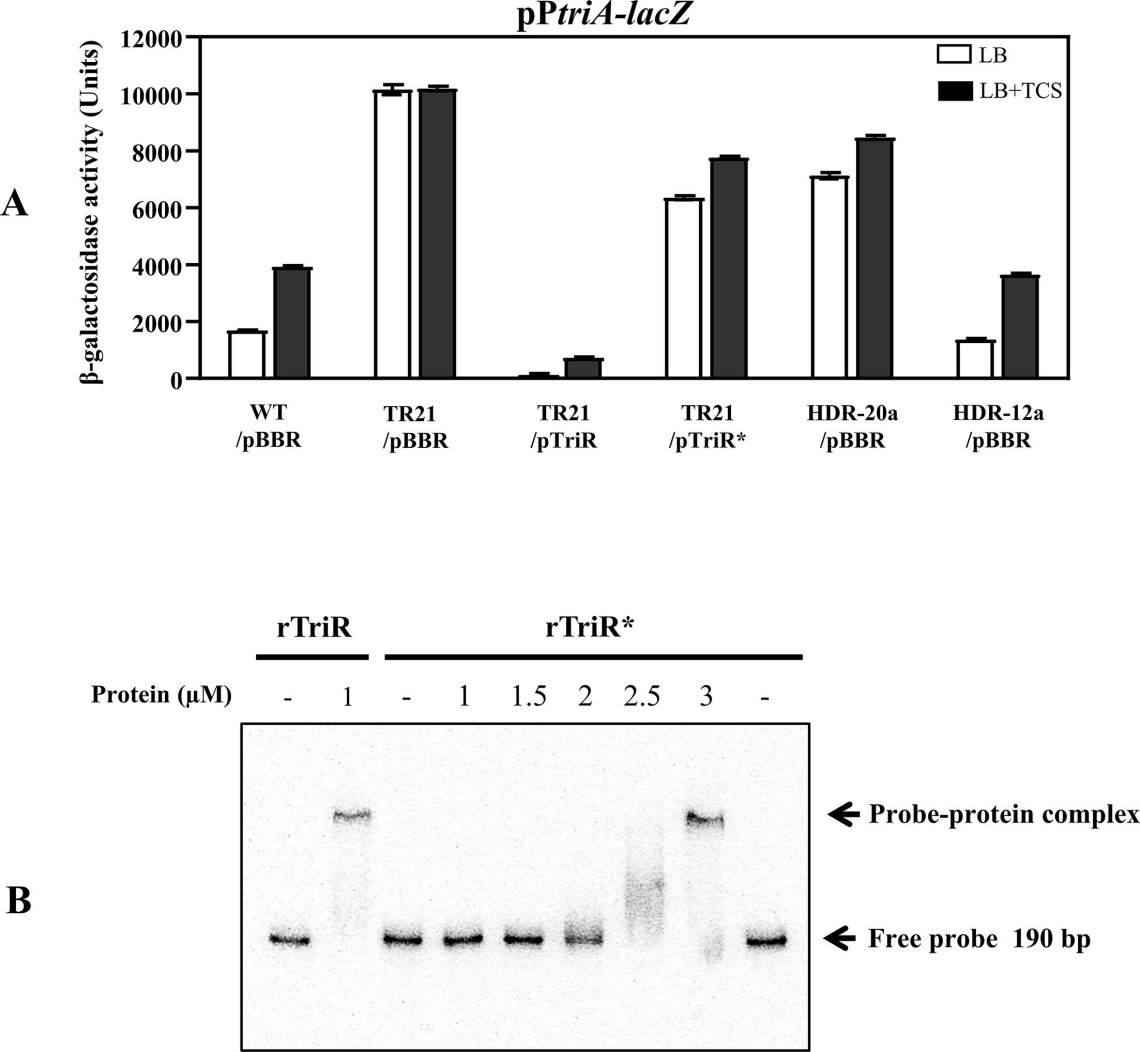
The mutated *triR* from HDR-20a has a defect in the repressor function due to the reduction in its DNA-binding activity. (**A**) The *triA* promoter-*lacZ* fusion assay. β-galactosidase activity was measured using exponential-phase cells harboring the pP*triA-lacZ* plasmid and grown in LB. For TCS treatment, cells were incubated with 5 µg/mL TCS for 30 min. Cells were WT, TR21, HDR-20a, and HDR-12a carrying either the plasmid vector pBBR, functional *triR* gene on the pTriR plasmid, or mutated *triR* gene (triR*, N157T mutation) on the pTriR* plasmid. The values of the β-galactosidase activities are the means of biological triplicates ± SD (bars). (**B**) EMSA was performed with a constant amount of ^32^P-labeled DNA fragment (190-bp probe containing a TriR binding site) and varying amounts (1, 1.5, 2, 2.5, and 3 µM) of rTriR and rTriR*. Bands corresponding to the free probe and the probe–protein complex are marked with arrows.

The *triA* promoter-*lacZ* fusion assay and electrophoretic mobility shift assay (EMSA) were performed to further assess the repressor function and DNA-binding ability of TriR*. The β-galactosidase (β-gal) activity of the *triA* promoter (pP*triA-lacZ*) was determined using WT, TR21, HDR-12a, and HDR-20a cells harboring pBBR, pTriR, or pTriR* (Fig. 4A). Similar to the previous study findings (26), the β-gal activity was higher in the *triR* mutant strain (TR21/pBBR, ~ 10013 units) than in the WT strain (WT/pBBR, ~ 1175 units), and complementation with the functional *triR* gene in *trans* via pTriR could fully suppress β-gal activity (TR21/pTriR, ~ 141 units) under the LB growth conditions. Unlike pTriR, pTriR* could partially repress β-gal activity (TR21/pTriR*, ~ 6106 units, LB) (Fig. 4A).

These results suggested that TriR* has a defective repressor function, as reflected by the high β-gal activity in HDR-20a/pBBR (~6180 units, LB) (Fig. 4A). HDR-12a/pBBR (~ 963 units, LB) showed similar β-gal activity to WT/pBBR (Fig. 4A). In the presence of 5 µg/mL TCS (LB + TCS, Fig. 4A), *triA-lacZ* fusion was induced in all the tested strains except TR21/pBBR (loss of *triR*). β-gal activity in TR21/pBBR was constitutively expressed at high levels in both LB and LB + TCS conditions, implying that TriR* (TR21/pTriR* and HDR-20a/pBBR) remained responsive to TCS (LB + TCS, Fig. 4A).

The purified recombinant proteins rTriR and rTriR* (with a short peptide consisting of eight amino acids, WSHPQFEK, fused in a frame to the C-terminus) were used in EMSAs to compare the DNA-binding abilities of the mutated TriR (rTriR*) and wild-type TriR (rTriR) proteins using constant amounts of the 190-bp DNA probe (*triA* promoter containing a TriR-binding site) (26). Figure 4B shows that the shifted band (DNA-protein interaction) was detected in the reaction containing 1 μM rTriR and the DNA probe. However, higher amounts of rTriR* (3 µM) were required to detect the shifted band. These results implied that rTriR* has a lower DNA-binding ability than rTriR, thereby explaining the observation that pTriR* could partially repress the β-gal activity of the *triA* promoter (Fig. 4A).

### Transcriptomic analysis of HDR-20a

**Fig 5:**
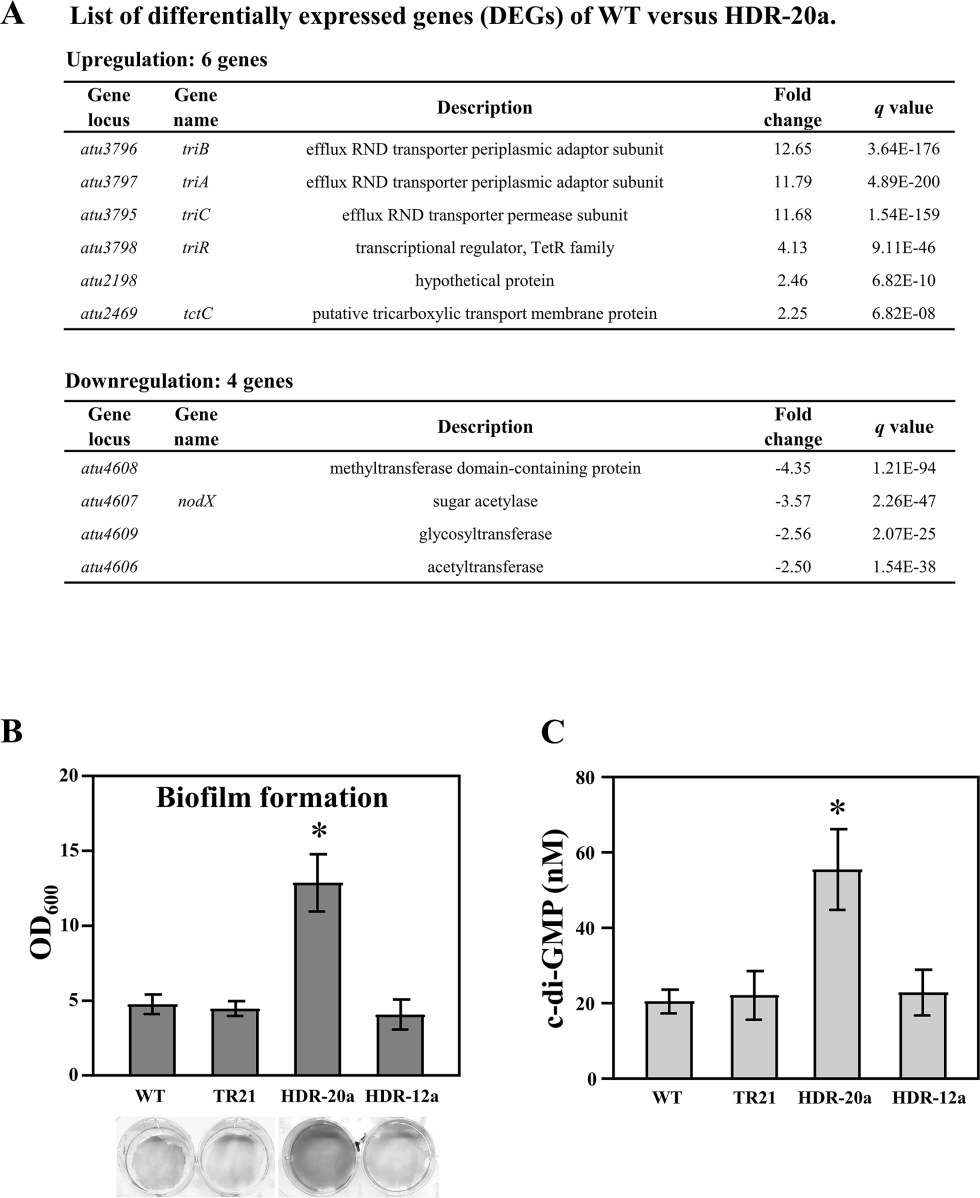
Transcriptomic analysis of HDR-20a and determination of biofilm formation and c-di-GMP levels. (**A**) List of differentially expressed genes (DEGs) in WT versus HDR-20a cells. Log_2_ fold change >1 or <-1, and *q* value <0.001 were used as a threshold. Biofilm formation (**B**) and c-di-GMP levels (**C**) in WT, TR21, HDR-12a, and HDR-20a cells. The results represent the means of triplicate independent samples ± SD. For comparison between WT and other strains, the bars marked with * are significantly different (*p* value < 0.05 in an unpaired Student’s *t*-test).

Mutation of the *triR* gene coding for the TriR transcription regulator was evaluated for its effects on transcriptomic alteration in HDR-20a cells. RNA-seq was used to determine the gene expression changes in HDR-20a compared with the WT strain, using the criteria of a fold change >2 indicating upregulated or downregulated genes and a *q* value <0.001, indicating differentially expressed genes (DEGs) (Fig. 5A). The DEGs were validated by qRT-PCR (Fig. S3). A total of six DEGs, including *triR*, *triABC* operon, *atu2198* (hypothetical protein), and *tctC* (putative tricarboxylic transport membrane protein), were considered upregulated genes in HDR-20a. A total of 4 DEGs, consisting of *atu4606* (acetyltransferase), *atu4607* (*nodX*, sugar acetylase), *atu4608* (methyltransferase domain-containing protein), and *atu4609* (glycosyltransferase), were identified as downregulated in HDR-20a. DNA sequence analysis did not reveal any potential TriR binding sites in the promoter regions of *atu2198, tctC,* or the *atu4606–atu4609* gene cluster, suggesting that TriR may not directly modulate the altered transcription of these genes.

### Increased biofilm formation and intracellular c-di-GMP in HDR-20a

The HDR-20a strain had a mutated *triR* (triR*) and exhibited higher TCS resistance (increased MIC value to TCS in Fig. 1B) but had lower *triA* expression (Fig. 3A) when compared with TR21 (loss of *triR*). Furthermore, HDR20a-TA (HDR-20a with *triA* disruption) was more resistant (~10^2^-fold) than TA20 (*triA* disruption) to 1 μg/mL TCS, as shown in Fig. 3B. These observations suggested that mechanisms other than the enhanced *triABC* expression may also contribute to the TCS-resistant phenotype of HDR-20a. Therefore, the possibility that bacterial biofilm formation could promote enhanced resistance to environmental stresses, including antibiotics and biocides, was explored.

Experiments using *E. coli* have shown that TCS exposure can interfere with fatty acid synthesis, leading to alterations in the membrane structure and increased biofilm formation and, in turn, conferring TCS tolerance (35). In the present study, biofilm formation was greater in HDR-20a cells than in WT, TR21, or HDR-12a cells (Fig. 5B). The intracellular signaling molecule c-di-GMP modulates the lifestyle switch from a unicellular, motile state to a sessile, multicellular, biofilm-associated state, and a correlation has been demonstrated between high c-di-GMP concentrations in the cell and biofilm formation in *A. tumefaciens* (36). The c-di-GMP levels were approximately 2.5-fold higher in HDR-20a cells than in WT, TR21, or HDR-12a cells (Fig. 5C), suggesting that the increased biofilm formation in HDR-20a may be due to elevated c-di-GMP levels.

### Transcriptomic analysis of HDR-12a

Unlike the expression observed in HDR-20a cells, the level of *triABC* expression in HDR-12a did not increase compared with that of WT cells (Fig. 3A). Genes that may be responsible for the TCS-resistant phenotype of HDR-12a were identified using RNA-seq to determine mRNA expression in HDR-12a versus WT cells. A total of 186 DEGs (146 upregulated and 40 downregulated genes) were identified based on fold change criteria of greater than 2-fold differences and *q* values <0.001 (Table S1). The upregulated DEGs (*aglE, cysD-2*, *cysH*, *cysK*, *dctP*, *dtcQ, fadB-2, fadD, frcC, hspL*, *hslV*, *mntH, rbsB-5*, *rpoH*, *sitA, ssuD*, *tctC*, and *troC*) and downregulated DEGs (*atu4447*, *cspA-4, cspA, dctA*, *mtlK*, *nirV*, and *norE*) were chosen (ranging from low [<2.5-fold], to medium [2.5- to 10-fold], and high [>10-fold] alterations), and their expression levels were validated by qRT-PCR (Fig. S4). The qRT-PCR results showed a similar trend for the expression of selected DEGs to that indicated by the RNA-seq data.

The DEGs of HDR-12a were functionally categorized based on the clusters of orthologous groups (COGs) (see Data Set S1). The largest COG category was “carbohydrate transport and metabolism,” followed by “amino acid transport and metabolism,” “posttranslational modification, protein turnover, chaperones,” “cell wall/membrane/envelope biogenesis,” “lipid transport and metabolism,” and “inorganic ion transport and metabolism” (Data Set S1). Overall, 24 DEGs fell into the group of functional unknowns and general function prediction only.

The most important biological metabolic pathways associated with DEGs were identified using KEGG pathway enrichment analysis (KOBAS-i 2.0) (Data Set S2). Significantly enriched (*p* value < 0.05) upregulated DEGs were associated with five pathways: sulfur metabolism, ABC transporters, monobactam biosynthesis, fatty acid degradation, and lysine degradation (Data Set S2). However, no significantly enriched pathway for downregulated DEGs was evident when a *p* value <0.05 was considered. A protein–protein interaction (PPI) network was also generated using STRING analysis (version 12.0) with a medium confidence score of 0.40 (Data Set S3). The upregulated DEGs were mapped into 17 PPI networks, in which the top pathways, based on gene count, were “sugar transport and metabolism,” “sulfur metabolism,” “stress responses,” “fatty acid metabolism,” “metal transporter,” “tripartite-type tricarboxylate (TTT) transporter,” and “tripartite ATP-independent periplasmic (TRAP) transporter.” The downregulated DEGs were mapped into 6 PPI networks, in which “ABC-type sugar transporter” and “nitrogen metabolism” were the top pathways.

**Fig 6:**
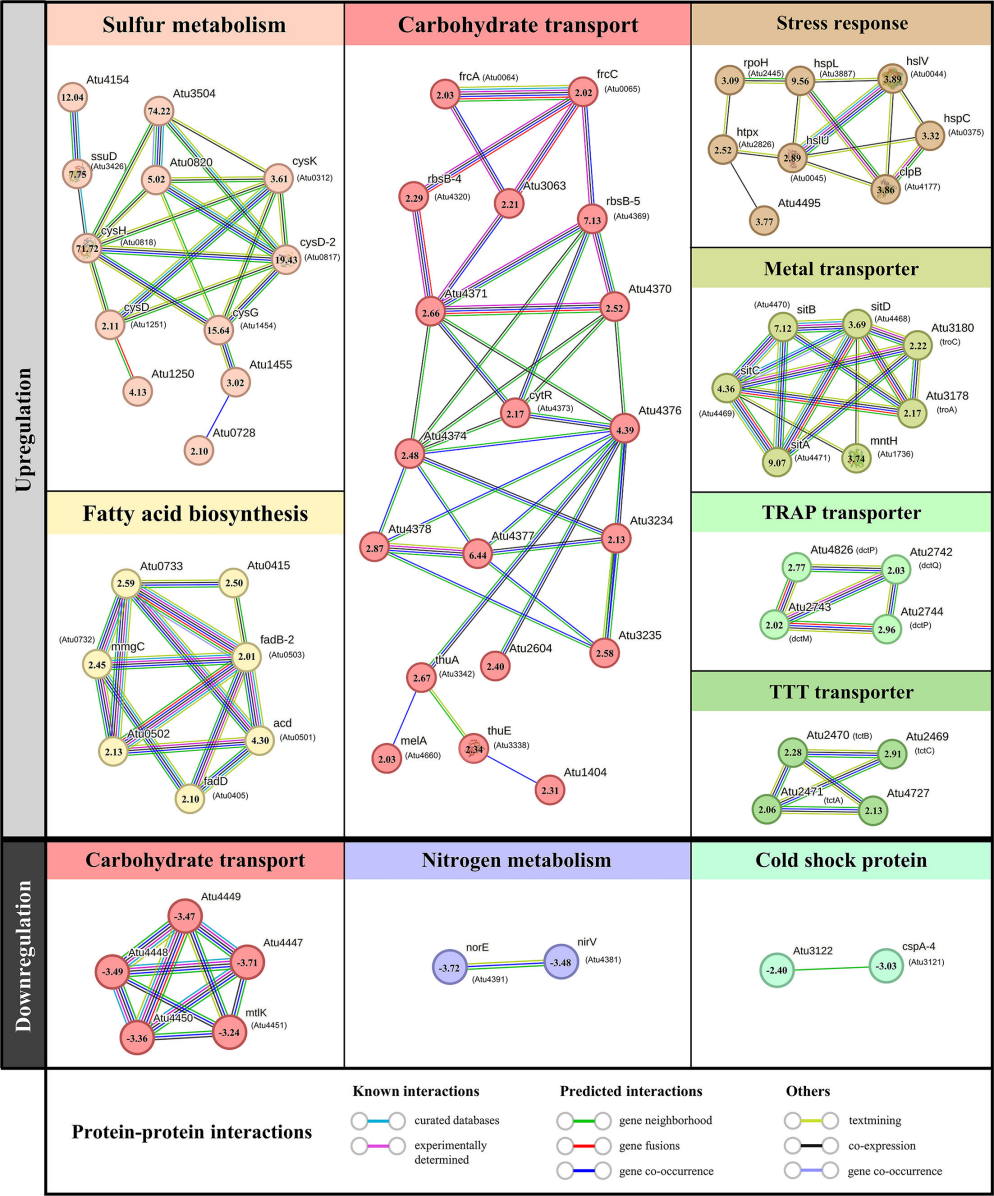
Protein–protein interaction network analysis of upregulated and downregulated DEGs of HDR-12a by STRING version 12.0. Only the main metabolic pathways are shown. Numbers inside each node indicate fold changes from RNA-seq analysis.

The COG, KEGG pathway enrichment analysis, and PPI network results revealed metabolic adaptation in HDR-12a (Fig. 6). Genes involved in sulfur metabolism, including *atu3504*, *cysH*, *cysD-2*, *cysG*, *atu4154,* and *ssuD*, were the most significantly enriched and highly upregulated (approximately 74-fold, 71-fold, 19-fold, 15-fold, 12-fold, and 7.7-fold upregulations, respectively). Among the upregulated ABC transporters, the *sitABCD* manganese-specific uptake system (*atu4468-atu4471* operon, upregulated approximately 3.6-fold to 9-fold) was the most highly enhanced. Other metal importers for manganese (*mntH*) and zinc (*troCBA*) also showed approximately 3.7-fold and 2-fold enhancements, respectively. Several DEGs encoding proteins related to carbohydrate transport and metabolism, including fructose (*frcA* and *frcC*), glucosides (*atu4377* and *atu4378*), and ribose (*atu4370*, *atu4371*, *rbsB-4,* and *rbsB-5*), were elevated approximately 2-fold to 7-fold. Expression of stress response genes (*clpB*, *hspC*, *hspL*, *hslU*, *hslV,* and *rpoH*) was elevated approximately 2.8-fold to 9.5-fold. Genes in pathways involving fatty acid metabolism (*atu0502*, *fadB-2,* and *fadD*), TTT transport (*atu2469-atu2470-atu2471* [*tctCBA*] and *atu4727* [*tctC*]), and the TRAP transporter (*atu2742-atu2743-atu2744* [*dctQMP*], and *atu4826* [*dctP*]) were increased approximately 2-fold to 3-fold. Among the downregulated DEGs, *dctA* (*atu3298*) encoding aerobic C4-dicarboxylate transport protein was the most reduced, by approximately 4-fold. Other downregulated DEGs (with reductions ranging from 2-fold to 3.7-fold) were related to ABC-type carbohydrate transporters (*atu4447*, *atu4448*, *atu4449*, *atu4450*, and *mtlK*), nitrogen metabolism (*norE* and *nirV*), and cold-shock proteins (*cspA, cspA-4*, and *atu3122*).

### Both HDR-12a and HDR-20a showed increased resistance to chloramphenicol and erythromycin

The possibility that the adaptive TCS-resistant strains could acquire cross-resistance to antibiotics was tested by determining the antibiotic susceptibility of HDR-12a and HDR-20a compared with the WT strain using Epsilometer test strips and disk diffusion assays (data not shown). The tested drugs included a β-lactam (meropenem), cephalosporins (ceftazidime, cefepime, ceftriaxone, and piperacillin), aminoglycosides (netilmicin, tobramycin, kanamycin, amikacin, and gentamicin), quinolones (ciprofloxacin, levofloxacin, and norfloxacin), macrolides (azithromycin, clarithromycin, and erythromycin), and others (minocycline, tetracycline, trimethoprim-sulfamethoxazole, chloramphenicol, colistin, polymyxin B, and fosfomycin). No differences in resistance were detected between HDR-12a and HDR-20a cells compared with WT cells for all the tested antibiotics except chloramphenicol and erythromycin. A plate sensitivity assay confirmed that both HDR-12a and HDR-20a showed similar phenotypes, as both were more resistant than WT cells to 30 μg/mL chloramphenicol (~10^2^-fold) and 20 μg/mL erythromycin (~10^3^-fold) (Fig. S5). Furthermore, *triA* disruption could not reverse the resistant phenotype of HDR-20a (compared HDR-20a with HDR20a-TA, Fig. S5), suggesting that chloramphenicol and erythromycin resistance were not due to the overproduction of the TriABC efflux pump. At present, the mechanisms underlying CHL and ERY resistance in HDR-12a and HDR-20a cells remain unclear.

## DISCUSSION

In the present study, *A. tumefaciens* served as a model soil bacterium for studying adaptation and the induction of resistance mechanisms in response to TCS exposure. At low levels, TCS targets the enoyl-acyl carrier protein reductase enzyme, thereby blocking bacterial type II fatty acid synthesis (8). Previous studies in *E. coli* (7) and *Salmonella enterica* serovar Typhimurium (37) have reported that amino acid substitutions within FabI were the primary mechanism conferring TCS resistance. However, FabI alone did not mediate high-level TCS resistance in *S. enterica*, as a subsequent investigation identified the AcrAB-TolC system as responsible for intrinsic and high-level resistance to TCS (37). Proteomic analysis in *S. enterica* further revealed a common metabolic TCS resistance network that included proteins involved in the production of pyruvate (which feeds fatty acid biosynthesis) or in alternative metabolic routes for generating fatty acids (38). In our study, no mutations were detected in the *fabI* homologs (*atu0149* and *atu0757*) of either of the two adaptive TCS-resistant strains. When exposed to the selective pressure conditions of high-dose TCS, HDR-20a evolved a mutation in the *triR* transcription regulator gene that resulted in *triABC* operon overexpression.

TriABC is a TCS-specific efflux pump and plays a dominant role in TCS resistance in *A. tumefaciens* (26). Disruption of fatty acid synthesis by TCS also results in alterations in the membrane structure and increased biofilm formation, which also confer TCS tolerance in *E. coli* (35). In the present study, transcriptomic analysis of HDR-20a revealed downregulation of a cluster of genes, including *atu4606* (acetyltransferase), *atu4607* (*nodX*, sugar acetylase), *atu4608* (methyltransferase), and *atu4609* (glycosyltransferase), which may be responsible for modification of the cell surface structure (39). The major lipid-bound carbohydrate component of the outer membrane of gram-negative bacteria is lipopolysaccharide (LPS), and modification of the O-antigen of LPS by adding extra moieties, such as glucosyl and acetyl groups, is crucial in bacterial interactions with the external environment (39). This raises the possibility that regulation of the *atu4606*, *atu4607*, *atu4608*, and *atu4609* genes might affect the structure of the outer membrane and thereby influence TCS susceptibility in HDR-20a cells.

Although increased c-di-GMP and biofilm formation may also contribute to TCS resistance in HDR-20a, the findings presented here indicate that a *triR* mutation that promotes TriABC overproduction appears to be the key mechanism responsible for the high TCS resistance. Conversely, the HDR-12a cells, which had a lower MIC than HDR-20a for TCS but lacked a *triR* mutation, may require coordinated changes in gene expression to develop TCS resistance, as a number of mechanisms may be involved and act in synergy to achieve TCS resistance.

TCS exposure was also found to lead to the development of cross-resistance to the antibiotics chloramphenicol and erythromycin, which inhibit protein synthesis by binding to the large ribosomal subunit and inhibiting the assembly of the large and small subunits (40, 41). Mutations in the 23S rRNA gene could confer resistance to both chloramphenicol and erythromycin (42). However, the WGS and transcriptomic analysis of HDR-12a and HDR-20a did not reveal any obvious alterations in the genes involved in chloramphenicol or erythromycin resistance. The *A. tumefaciens atu4738* (*cat*) gene encodes chloramphenicol acetyltransferases, which can inactivate chloramphenicol (43). Whether the CAT activity of the TCS adaptive strains might have increased requires further investigation. Moreover, there is the possibility that TCS drove the emergence of antibiotic resistance phenotypes in HDR-12a and HDR-20a through shared mechanisms. The WGS analysis revealed that both adaptive TCS-resistant strains might have a defect in an ABC transporter (of unknown function) due to a frameshift deletion in *atu4668* (ABC-2 type transport system permease protein, 372 aa). DNA sequence analysis suggested that *atu4668* forms an operon with *atu4666* (HlyD family secretion protein, 354 aa) and *atu4667* (nucleotide-binding/ATPase protein, 925 aa). Therefore, characterizing the function of the *atu4666-atu4667-atu4668* operon would be of interest in a future study, as would determining whether this putative ABC-type 2 transporter is responsible for the phenotype of the adapted strains.

Previous studies in other bacteria have indicated that resistance to TCS, chloramphenicol, and erythromycin might reflect an alteration in some outer membrane and LPS structure (32, 44). The expression of the *tctC* (*atu2469*) gene increased approximately 2-fold in both HDR-12a and HDR-20a cells. TctC was first found to bind citrate and mediate its uptake in *Salmonella* Typhimurium (45). Increased citrate uptake may lead to enhanced acetyl-CoA production to support fatty acid synthesis and cell surface structural modifications required for TCS and antibiotic tolerance in HDR-12a and HDR-20a. Moreover, TctC homologs have been reported to play roles in regulatory processes and signaling (44, 46). Further investigation is needed to test whether upregulation of TctC may confer TCS and antibiotic resistance in *A. tumefaciens*.

One limitation of the present study was that it focused on only two adaptive strains of *A. tumefaciens* exposed to high (sub-MIC) TCS doses. To gain a better understanding of the potential environmental risks posed by TCS, further investigations should be performed with low-dose TCS exposures that reflect environmentally relevant concentrations. Although our WGS and transcriptomic analyses have provided insights into the mechanisms that drive TCS resistance, the inclusion of proteomic and metabolomic approaches would help further identify the proteins and metabolic pathways involved in the development of TCS and antibiotic resistance.

In conclusion, adaptive laboratory evolution of *A. tumefaciens* revealed different mechanisms of resistance in adaptive strains to high-dose TCS exposure. The two isolated strains, HDR-12a and HDR-20a, exhibited higher MIC values (20 and 32 μg/mL, respectively) against TCS when compared with the WT strain (10 μg/mL). Proper functioning of the transcriptional repressor TriR was crucial for controlling the expression of the *triABC* operon, which encodes the specific TCS efflux pump. A single base change in the *triR* gene led to the malfunction of TriR and the overexpression of the TriABC efflux pump, which plays a dominant role in conferring high resistance to TCS in HDR-20a cells. Increased c-di-GMP and biofilm formation may also confer TCS resistance in HDR-20a. Conversely, in HDR-12a cells, the transcriptomic adaptations of several genes associated with ABC transporters and the metabolism of sulfur, fatty acids, and carbohydrates may play roles in the development of TCS resistance. The finding that both HDR-12a and HDR-20a exhibit resistance to chloramphenicol and erythromycin reinforces the link between TCS exposure and the emergence of antibiotic resistance.

## MATERIALS AND METHODS

### Bacterial strains, plasmids, and DNA manipulations

Table 1 shows the bacterial strains and plasmids used in this study. *A. tumefaciens* and *Escherichia coli* were grown at 28°C and 37°C, respectively, in Luria-Bertani (LB) broth or LB containing 1.5% agar (LA) as described previously (26). Overnight bacterial cultures were subcultured into fresh LB medium to yield an optical density of 0.1 at 600 nm (OD_600_). The cells were incubated for another 4 h to reach an OD_600_ of 0.5, when the cells were considered to be in the exponential growth phase. DNA manipulations were performed according to standard protocols (47). Table S2 shows the primers used in this study. DNA sequencing was performed by ATGC Co., Ltd. (Thailand).

**TABLE 1:** Bacterial strains and plasmids used in this study^*a*^

Strain or plasmid	Genotype or characteristics	Reference or source
*A. tumefaciens* strains		
NTL4	Wild-type (WT) strain, a Ti plasmid-cured derivative of strain C58	48
TA20	*triA* mutant, *atu3797*::pKNOCK-Km, Km^r^	26
TR21	*triR* mutant, *atu3798*::pKNOCK-Gm, Gm^r^	26
HDR-12a	An adaptive triclosan-resistant strain	This study
HDR-20a	An adaptive triclosan-resistant strain	This study
HDR20a-TA	HDR-20a with *triA* disruption (*atu3797*::pKNOCK-Km), Km^r^	This study
*E. coli* strains		
BW20767	Host for plasmids pKNOCK-Gm and pKNOCK-Km	49
DH5α	Host for general DNA cloning	50
Plasmids for gene inactivation		
pKNOCK-Km	Suicide vector, Km^r^	51
pKNOCKTRIA	Internal coding region of *triA* cloned into pKNOCK-Km, Km^r^	26
Plasmids for complementation		
pBBR1MCS-4	Expression vector, Ap^r^ (pBBR)	52
pTriR	Full-length of wild-type *triR* cloned into pBBR1MCS-4, Ap^r^	26
pTriR*	Full-length of mutated *triR* (N157T) cloned into pBBR1MCS-4, Ap^r^	This study
Plasmids for protein expression and purification		
pASK-IBA3	Protein expression vector, Ap^r^	IBA
pTriR-Strep-tag	Coding region of wild-type *triR* cloned into pASK-IBA3, Ap^r^	This study
pTriR*-Strep-tag	Coding region of mutated *triR* (N157T) cloned into pASK-IBA3, Ap^r^	This study
Plasmids for promoter-*lacZ* fusions		
pPR9TT	Broad-host range vector carries a promoterless *lacZ* gene, Ap^r^	53
pP*triA-lacZ*	368 bp *triA* promoter region containing a TriR box cloned into pPR9TT, Ap^r^	26

^*a*^ Ap^r^, ampicillin resistance; Gm^r^, gentamicin resistance; Km^r^, kanamycin resistance.

### Adaptive laboratory evolution protocol for selecting HDR-12a and HDR-20a strains with enhanced TCS tolerance

A 5 mM TCS (Sigma-Aldrich-C7727) stock solution was prepared in dimethyl sulfoxide (DMSO). The adaptive resistance strains were isolated through exposure to gradually increasing concentrations of TCS (8, 12, 16, and 20 µg/mL). The wild-type (WT) *A. tumefaciens* strain NTL4 was streaked onto an LA plate and incubated at 28°C for 48 h. A single colony of the WT strain was inoculated in 8 mL of LB broth and grown at 28°C with shaking for 18 h. The cell culture was then serially diluted tenfold in 1 mL of fresh LB, and 100 µL of a 10^−5^ dilution was spread on an LA plate containing 8 µg/mL of TCS (LA-TCS8). The plate was incubated at 28°C for 48 h, and the surviving colonies on the LA-TCS8 plate were defined as high-dose resistant-8 (HDR-8). One colony from the LA-TCS8 plate was inoculated in fresh LB broth containing 8 µg/mL TCS (LB-TCS8) and grown at 28°C with shaking for 18 h. The cell culture was then diluted in fresh LB and spread on an LA plate containing 12 µg/mL of TCS (LA-TCS12). One surviving colony from the LA-TCS12 plate was inoculated in LB-TCS12 for subsequent selection of HDR-16 and HDR-20 on LA plates containing 16 and 20 µg/mL of TCS, respectively. The two adaptive TCS-resistant strains, named HDR-12a and HDR-20a, were single colonies picked from the LA-TCS12 and LA-TCS20 plates, respectively. HDR-12a and HDR-20a were grown in LB for 18 h and then preserved at −70°C in 15% glycerol for further investigation.

### Construction of HDR20a-TA strain (HDR-20a with *triA* disruption)

The *triA* gene was disrupted using the insertional gene inactivation method (26). The pKNOCKTRIA plasmid containing the internal coding region of *triA* (26) was transferred to HDR-20a by conjugation. The HDR20a-TA strain was selected on an LA plate containing 30 µg/mL kanamycin and then verified using southern blot analysis.

### Construction of plasmids pTriR and pTriR* for complementation

The pTriR plasmid was generated previously (26) by cloning full-length *triR* (621 bp) without a native promoter into either the pBBR1MCS-2 or pBBR1MCS-4 plasmid expression vector (52). A similar approach was used here to construct the pTriR* plasmid. The mutated *triR* gene (triR*, N157T mutation) was amplified by polymerase chain reaction (PCR) with primers BT7874 and BT7875 using genomic HDR-20a DNA as a template and was inserted into either pBBR1MCS-2 or pBBR1MCS-4 at the *Sma*I site. DNA sequencing confirmed the DNA cloning sequences.

### Plate sensitivity assay and determination of the minimum inhibitory concentration (MIC)

The agar dilution method was performed as previously described (26). Exponential growth phase cells grown in LB were adjusted to an OD_600_ of 0.125. Ten-fold serial dilutions were made in LB, and 10 µL of each dilution was spotted on LA or LA containing various TCS concentrations (1–32 μg/mL in 2 μg/mL increments). TCS was dissolved in DMSO, and the control was an LA plate containing an equal amount of DMSO. After incubation at 28°C for 48 h, the MIC (defined as the lowest TCS concentration that inhibited visible bacterial growth) was determined. The experiment was repeated at least twice to ensure result reproducibility.

### Quantitative real-time PCR (qRT-PCR) determination of gene expression

The qRT-PCR was performed as described previously (26) using exponential-phase cells grown in 20 mL of LB. The cell pellet was suspended in 300 µL of 0.3 M sucrose containing 10 mM sodium acetate (NaOAc). A lysis buffer (300 µL; 2% sodium dodecyl sulfate and 10 mM NaOAc) was added, and the mixture was incubated at 65°C with gentle mixing. Hot phenol (300 µL), maintained at 65°C, was added. After incubation at 65°C for 5 min with occasional mixing, the phases were separated by centrifugation at 12,000 rpm for 5 min. The aqueous phase was reextracted twice with 300 µL of hot phenol, followed by two extractions with chloroform (300 µL). RNA was precipitated by adding 3 M NaOAc (at 10% of the volume) and two volumes of absolute ethanol. After incubation at 70 °C for 1 h, the RNA was pelleted by centrifugation at 12,000 rpm for 10 min and washed once with 500 µL of 70 % ethanol. After drying, the RNA pellet was suspended in diethylpyrocarbonate (DEPC)-treated sterile distilled water. The RNA samples were treated with DNase I, RNase-free (Thermo Scientific), and reverse-transcribed using RevertAid Reverse Transcriptase (Thermo Scientific) with random hexamer primers (BioDesign, Thailand). Specific RNA transcripts were detected using the specific primers (Table S1). The expression of the gene of interest was normalized to that of 16S rRNA (a housekeeping control gene). The relative changes in gene expression were calculated using the 2^-ΔΔCt^ method (54).

### WGS and data analysis

Genomic DNA was isolated from overnight LB cell cultures of WT, HDR-12a, and HDR-20a cells using the standard protocol (47). Each cell culture (1.5 mL) was collected and resuspended in 100 µL of 1× TE buffer (20 mM Tris-HCl, pH 8.0, and 2 mM EDTA). The cells were lysed with 1× lysis buffer (40 mM Tris-HCl, pH 7.8, 20 mM NaOAc, pH 5.2, 1 mM EDTA, and 1% SDS), then 5 M NaCl (500 µL) was added. The DNA was precipitated by combining 450 µL of the clear supernatant with 1 mL of chilled absolute ethanol and then collected by spooling, washed with 500 µL of 70% ethanol, air dried, and resuspended in 100 µL of 1× TE buffer. The genomic DNA quality was assessed using a NanoDrop spectrophotometer and gel electrophoresis.

WGS was performed by Vishuo Biomedical (Thailand) Ltd. A total of 200 μg of DNA per sample was fragmented to an average size of 300–350 bp using Covaris Adaptive Focused Acoustics technology. The fragmented DNA was treated with an End Prep Enzyme Mix for end repair, 5' phosphorylation, and 3' adenylation, followed by adapter ligation to both ends. The adapter-ligated DNA size was selected using DNA cleanup beads and then PCR-amplified for eight cycles using P5 and P7 primers. The PCR products were purified, validated using an Agilent 2100 Bioanalyzer, and sequenced on an Illumina NovaSeq 6000 System with 150 bp paired-end reads. Data analysis included sequence trimming with fastp (v0.23.0) to remove adapters, low-quality bases, and PCR artifacts. Clean reads were mapped to the reference genome (ASM9202v1) using Sentieon (v202112.02), followed by duplicate removal and variant calling for SNVs and InDels. Variants were annotated using ANNOVAR (v21 Apr 2018), and structural variations were analyzed using BreakDancer and CNVnator. Genome annotation was performed using the Kyoto Encyclopedia of Genes and Genomes (KEGG) database of *A. tumefaciens* C58 (https://www.genome.jp/kegg) (55).

### The *triA* promoter-*lacZ* fusion and β-galactosidase activity assay

Exponential-phase cells harboring the pP*triA-lacZ* plasmid (26) were grown in LB. For TCS treatment, cells were incubated with 5 µg/mL TCS for 30 min. Cells were then washed twice with 1× phosphate-buffered saline (PBS). Harvested cells were adjusted to an OD_600_ of 1 in 900 µL of 1× PBS. The cells were permeabilized at 28°C for 5 min with 0.1% SDS (50 µL) and chloroform (50 µL). β-Galactosidase activity was measured as described previously (26). Aliquots (50 µL) of the permeabilized cells were incubated with 850 µL of the ortho-nitrophenyl-β-galactoside (ONPG) substrate solution (3.3 mM ONPG, 1 mM MgCl_2_, and 100 mM 2-mercaptoethanol in 1× PBS, pH 7.4). After incubation at 28°C for 5 min, 2 M Na_2_CO_3_ (100 µL) was added to stop the reaction. The OD_420_ was measured, and units of β-galactosidase activity were calculated as the change in OD_420_ min^−1^ per OD_600_ of culture. The results were reported as the means of three biological replicates ± standard deviation (SD).

### Protein purification and EMSA

The pTriR-Strep-tag plasmid was constructed and used to produce the recombinant TriR protein (rTriR, a short peptide consisting of eight amino acids, WSHPQFEK, fused in frame to the C-terminus) (26). To generate the pTriR*-Strep-tag plasmid for producing rTriR*, the coding region of the mutated *triR* was amplified (with BT7880 and BT7881, which contained *Bsa*I sites) using genomic HDR-20a DNA and inserted into pASK-IBA3 at the *Bsa*I site (IBA, Germany). The rTriR and rTriR* proteins were overproduced in *E. coli* DH5α and purified using a Strep-Tactin Sepharose column, as described previously (26). The purified proteins were analyzed using SDS-PAGE and Coomassie blue staining (Fig. S6). The DNA probe, end-labeled with ^32^P (190 bp fragment containing the TriR box 5′-TTGACTATTC-GGTTAGTCAA-3′), was prepared, and EMSA was performed as previously described (26). The binding reactions contained the same amount of DNA probe and varying concentrations of purified proteins, as indicated in the figure legend.

### RNA sequencing and transcriptomic data analysis

Exponential-phase WT, HDR-12a, and HDR-20a cells grown in 10 mL of LB were harvested. Total RNA samples were extracted from three independent biological replicates of each strain using the RNeasy Protect Bacteria Mini Kit (Qiagen) following the manufacturer’s protocol. The RNA was purified with VAHTS RNA clean beads (Vazyme #N412, Cellagen Technology, USA), and its quality and quantity were determined using an Agilent 2100/2200 Bioanalyzer (Agilent Technologies, Palo Alto, California, USA). The rRNA depletion, cDNA library preparation, and paired-end 150 nucleotide length sequencing on the Illumina Hiseq platform were performed by Vishuo Biomedical (Thailand) Ltd., as described previously (56). Genes with a false discovery rate (*q* value) <0.001 and a log_2_ fold change >1 or <−1 (i.e., a fold change >2 in upregulated and downregulated genes) were considered differentially expressed genes (DEGs) between the WT and tested strains. The qRT-PCR was performed as previously described (26) to confirm the expression changes in the selected DEGs. Clusters of orthologous genes (COG) and the KEGG orthology (KO) databases were used to assign possible functions to the DEGs. KEGG pathway enrichment analysis was performed using KOBAS-i 2.0 (57), and pathways were considered significantly enriched if they had a *p* value less than 0.05. A protein–protein interaction (PPI) network was generated using STRING version 12.0 (58) with a medium confidence score of 0.40.

### Crystal violet biofilm formation assay

Biofilm formation was measured using crystal violet staining (59). Exponential-phase cells grown in LB were adjusted to yield OD_600_ 0.1. A 2 mL sample of the cell suspension was transferred to each well of a 6-well polystyrene plate (Nunclon Delta Surface, Thermo Scientific) and incubated without shaking at 28°C for 18 h. The cell suspension was removed, the plate was washed twice with distilled water (2 mL), and 1% crystal violet (1 mL) was added to each well and incubated at room temperature for 30 min. After washing twice with distilled water (2 mL), the biofilm layer was solubilized with 95% ethanol (1 mL), and the OD_600_ was measured using 95% ethanol as a blank. Each bacterial strain was tested in triplicate, and the experiment was repeated at least three times to ensure result reproducibility.

### Quantification of intracellular c-di-GMP levels 

The c-di-GMP levels in the WT, HDR-12a, HDR-20a, and TR21 strains were measured using a Cyclic-di-GMP Assay Kit (Lucerna, USA). Overnight cell cultures grown in LB (10 mL) were pelleted and washed twice with sterile Milli-Q water (5 mL). The cells were adjusted to an OD_600_ of 0.35 in sterile Milli-Q water. A standard curve was generated by serially diluting the c-di-GMP standard to final concentrations of 0, 20, 40, 60, 80, and 100 nM. The adjusted cells (20 µL) or standard solutions were added to the assay reactions according to the manufacturer’s instructions. Reactions were run in a 96-well flat-bottom black plate (PerkinElmer OptiPlate). After incubation for 30 min in the dark at room temperature, the fluorescence intensity was measured using a VICTOR Nivo Multimode Plate Reader (excitation 480/30 nm and emission 530/30 nm). The c-di-GMP concentrations were calculated based on the standard curve. The results are reported as the means of three biological replicates ± SD.

## Data Availability

WGS data and raw sequence RNA-Seq data are available from the NCBI Sequence Read Archive database under the BioProject accession numbers PRJNA1225026 and PRJNA1226920, respectively.

## SUPPLEMENTAL MATERIAL

Data Set S1 aem.01232-25-s0001.pdf COGs.

Data Set S2 aem.01232-25-s0002.pdf KEGG pathway enrichment analysis.

Data Set S3 aem.01232-25-s0003.pdf PPI network analysis.

Supplemental figures aem.01232-25-s0004.pdf Figures S1 to S6.

Supplemental tables aem.01232-25-s0005.pdf Tables S1 and S2.

ASM does not own the copyrights to Supplemental Material that may be linked to, or accessed through, an article. The authors have granted ASM a non-exclusive, world-wide license to publish the Supplemental Material files. Please contact the corresponding author directly for reuse.
