# Supplementary material for: Characterization of adaptive evolution strains for the development of triclosan resistance in Agrobacterium tumefaciens C58
Source: Appl Environ Microbiol. 2026 Jan 6;92(1):e01232-25. doi: 10.1128/aem.01232-25 (PMC12838394; doi:10.1128/aem.01232-25)
Supplement: Data Set S1 — COGs. [file aem.01232-25-s0001.pdf]

Data Set 1 Clusters of Orthologous Groups (COGs) analysis. Functional annotation of the differentially expressed genes (DEGs) between WT and HDR-12a was assigned according to COG categories using the NCBI database (https://www.ncbi.nlm.nih.gov/research/cog/). Numbers of downregulated (black bars) and upregulated (gray bars) DEGs in each COG category are indicated.

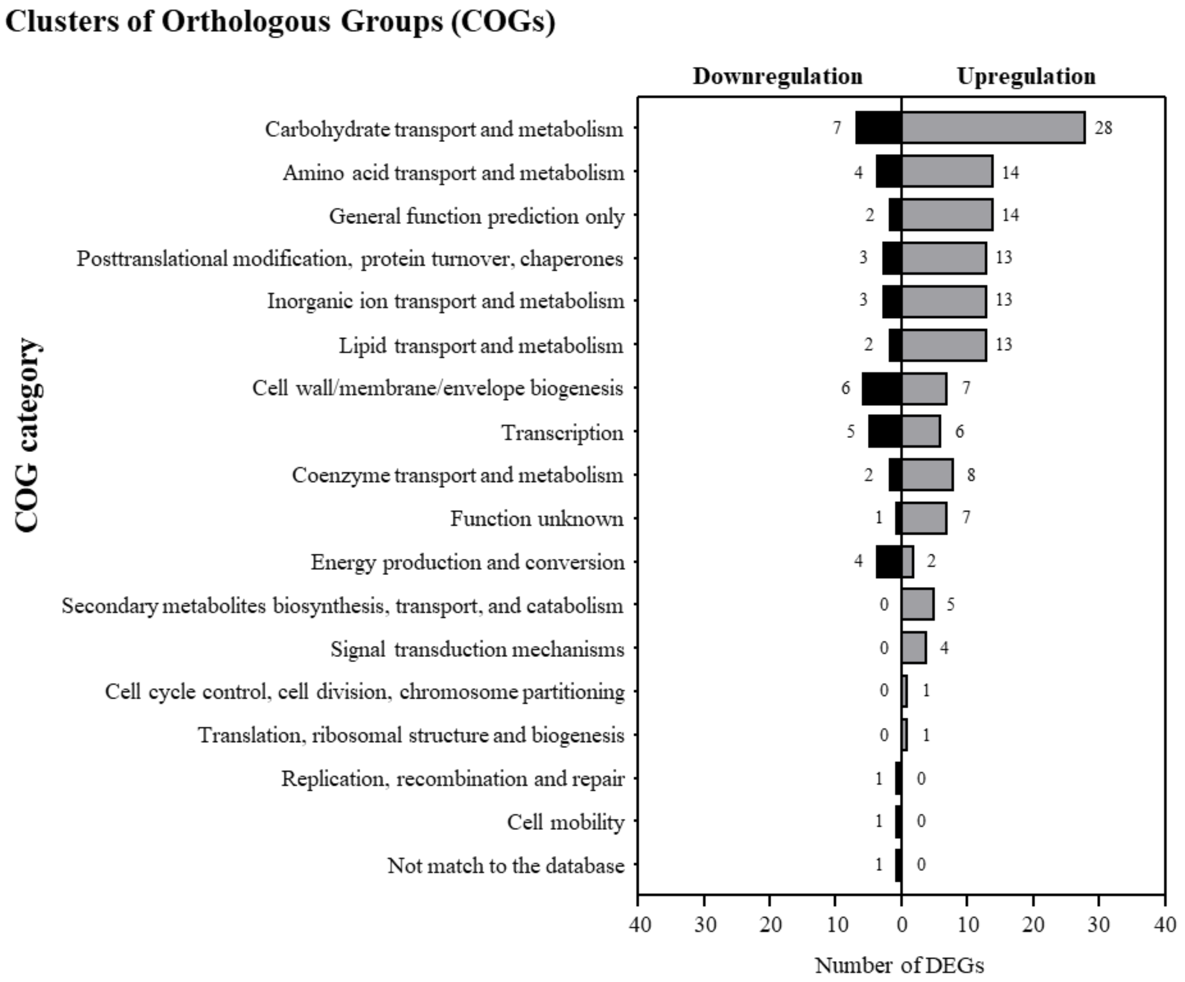

Upregulation: 146 DEGs

1. Metabolism

Gene	Gene name	COG ID	Annotation
**COG category C: Energy production and conversion**			
atu2469	*tctC*	COG3181	Tripartite-type tricarboxylate transporter, extracytoplasmic receptor component TctC
atu4727		COG3181	Tripartite-type tricarboxylate transporter, extracytoplasmic receptor component TctC
**COG category E: Amino acid transport and metabolism**			
atu0312	*cysK*	COG0031	Cysteine synthase
atu0817	*cysD-2*	COG0175	Phosphoadenylyl sulfate (PAPS) reductase/FAD synthetase or related enzyme
atu0818	*cysH*	COG0175	Phosphoadenylyl sulfate (PAPS) reductase/FAD synthetase or related enzyme
atu0946		COG1748	Saccharopine dehydrogenase, NADP-dependent
atu1251	*cysD*	COG2873	O-acetylhomoserine/O-acetylserine sulfhydrylase, pyridoxal phosphate-dependent
atu2757	*glnK*	COG0347	Nitrogen regulatory protein PII
atu3357		COG0834	ABC-type amino acid transport/signal transduction system, periplasmic component/domain
atu3358		COG0765	ABC-type amino acid transport system, permease component
atu4007	*arcA*	COG0010	Arginase/agmatinase family enzyme
atu4421		COG0687	Spermidine/putrescine-binding periplasmic protein
atu4769		COG1878	Kynurenine formamidase
atu5067	*dapA*	COG0329	4-hydroxy-tetrahydrodipicolinate synthase/ N-acetylneuraminate lyase
atu5071	*dppA*	COG0747	ABC-type transport system, periplasmic component
atu5394	*dapA*	COG0329	4-hydroxy-tetrahydrodipicolinate synthase/N-acetylneuraminate lyase
**COG category G: Carbohydrate transport and metabolism**			
atu0064	*frcC*	COG1172	Ribose/xylose/arabinose/galactoside ABC-type transport system, permease component
atu0065	*frcA*	COG1129	ABC-type sugar transport system, ATPase component
atu0591	*aglE*	COG1653	ABC-type glycerol-3-phosphate transport system, periplasmic component
atu0592	*aglF*	COG1175	ABC-type sugar transport system, permease component
atu0593	*aglG*	COG0395	ABC-type glycerol-3-phosphate transport system, permease component
atu1404		COG1653	ABC-type glycerol-3-phosphate transport system, periplasmic component
atu2696		COG2220	L-ascorbate lactonase UlaG, metallo-beta-lactamase superfamily
atu2742	*dctQ*	COG3090	TRAP-type C4-dicarboxylate transport system, small permease component YiaM

Gene	Gene name	COG ID	Annotation
**COG category G: Carbohydrate transport and metabolism**			
atu2743	*dctM*	COG1593	TRAP-type C4-dicarboxylate transport system, large permease component
atu2744	*dctP*	COG1638	TRAP-type C4-dicarboxylate transport system, periplasmic component
atu3063		COG1879	ABC-type sugar transport system, periplasmic component, contains N-terminal xre family HTH domain
atu3165		COG1653	ABC-type glycerol-3-phosphate transport system, periplasmic component
atu3235		COG1082	Sugar phosphate isomerase/epimerase
atu3338	*thuE*	COG1653	ABC-type glycerol-3-phosphate transport system, periplasmic component
atu3342	*thuA*	COG4813	Trehalose utilization protein
atu3891		COG1653	ABC-type glycerol-3-phosphate transport system, periplasmic component
atu3893		COG0395	ABC-type glycerol-3-phosphate transport system, permease component
atu4037	*gcd*	COG2133	Glucose/arabinose dehydrogenase, beta-propeller fold
atu4135	*gcd-2*	COG4993	Glucose dehydrogenase, PQQ-dependent
atu4320	*rbsB-4*	COG1879	ABC-type sugar transport system, periplasmic component, contains N-terminal xre family HTH domain
atu4369	*rbsB-5*	COG1879	ABC-type sugar transport system, periplasmic component, contains N-terminal xre family HTH domain
atu4370		COG1129	ABC-type sugar transport system, ATPase component
atu4371		COG1172	Ribose/xylose/arabinose/galactoside ABC-type transport system, permease component
atu4374		COG1082	Sugar phosphate isomerase/epimerase
atu4376		COG1082	Sugar phosphate isomerase/epimerase
atu4654		COG1653	ABC-type glycerol-3-phosphate transport system, periplasmic component
atu4660	*melA*	COG1486	Alpha-galactosidase/6-phospho-beta-glucosidase, family 4 of glycosyl hydrolase
atu4826	*dctP*	COG1638	TRAP-type C4-dicarboxylate transport system, periplasmic component
**COG category H: Coenzyme transport and metabolism**			
atu0817	*cysD-2*	COG0175	Phosphoadenylyl sulfate (PAPS) reductase/FAD synthetase or related enzyme
atu0818	*cysH*	COG0175	Phosphoadenylyl sulfate (PAPS) reductase/FAD synthetase or related enzyme
atu1454	*cysG*	COG0007	Uroporphyrinogen-III methylase (siroheme synthase)
atu2307		COG2141	Flavin-dependent oxidoreductase, luciferase family (includes alkanesulfonate monooxygenase SsuD and methylene tetrahydromethanopterin reductase)
atu3426	*ssuD*	COG2141	Flavin-dependent oxidoreductase, luciferase family (includes alkanesulfonate monooxygenase SsuD and methylene tetrahydromethanopterin reductase)
atu3726		COG2141	Flavin-dependent oxidoreductase, luciferase family (includes alkanesulfonate monooxygenase SsuD and methylene tetrahydromethanopterin reductase)
atu4097	*nadB*	COG0029	Aspartate oxidase
atu4098	*nadA*	COG0379	Quinolinate synthase

Gene	Gene name	COG ID	Annotation
**COG category I: Lipid transport and metabolism**			
atu0405	*fadD*	COG0318	O-succinylbenzoic acid-CoA ligase MenE or related acyl-CoA synthetase (AMP-forming)
atu0501	*acd*	COG1960	Acyl-CoA dehydrogenase related to the alkylation response protein AidB
atu0502		COG0183	Acetyl-CoA acetyltransferase
atu0503	*fadB-2*	COG1024	Enoyl-CoA hydratase/carnithine racemase
		COG1250	3-hydroxyacyl-CoA dehydrogenase
atu0649	*cfa*	COG2230	Cyclopropane fatty-acyl-phospholipid synthase and related methyltransferases
atu0732	*mmgC*	COG1960	Acyl-CoA dehydrogenase related to the alkylation response protein AidB
atu0733		COG1024	Enoyl-CoA hydratase/carnithine racemase
atu1338	*fabG-3*	COG1028	NAD(P)-dependent dehydrogenase, short-chain alcohol dehydrogenase family
atu1407		COG1028	NAD(P)-dependent dehydrogenase, short-chain alcohol dehydrogenase family
atu2611		COG0671	Membrane-associated phospholipid phosphatase
atu2679		COG2755	Lysophospholipase L1 or related esterase. Includes spore coat protein LipC/YcsK
atu3498		COG1012	Acyl-CoA reductase or other NAD-dependent aldehyde dehydrogenase
atu4377		COG2303	Choline dehydrogenase or related flavoprotein
**COG category P: Inorganic ion transport and metabolism**			
atu0820		COG1613	ABC-type sulfate transport system, periplasmic component
atu1736	*mntH*	COG1914	Mn^{2+} or Fe^{2+} transporter, NRAMP family
atu3178	*troA*	COG0803	ABC-type Zn uptake system ZnuABC, Zn-binding component ZnuA
atu3180	*troC*	COG1121	ABC-type Mn^{2+}/Zn^{2+} transport system, ATPase component
atu3504		COG4150	ABC-type sulfate transport system, periplasmic component
atu4136		COG3685	Iron homeostasis protein YciF (ferroxidase)
atu4154		COG0715	ABC-type nitrate/sulfonate/bicarbonate transport system, periplasmic component
atu4468	*sitD*	COG1108	ABC-type Mn^{2+}/Zn^{2+} transport system, permease component
atu4469	*sitC*	COG1108	ABC-type Mn^{2+}/Zn^{2+} transport system, permease component
atu4470	*sitB*	COG1121	ABC-type Mn^{2+}/Zn^{2+} transport system, ATPase component
atu4471	*sitA*	COG0803	ABC-type Zn uptake system ZnuABC, Zn-binding component ZnuA
atu4648	*betC*	COG3119	Arylsulfatase A or related enzyme, AlkP superfamily
atu5494		COG3685	Iron homeostasis protein YciF (ferroxidase)
**COG category Q: Secondary metabolites biosynthesis, transport, and catabolism**			
atu0867		COG0656	Aldo/keto reductase, related to diketogulonate reductase
atu2680		COG4181	Predicted ABC-type transport system involved in lysophospholipase L1 biosynthesis, ATPase component
atu5268	*dctP*	COG4663	TRAP-type mannitol/chloroaromatic compound transport system, periplasmic component
atu5269	*dctM*	COG4664	TRAP-type mannitol/chloroaromatic compound transport system, large permease component
atu5270	*dctQ*	COG4665	TRAP-type mannitol/chloroaromatic compound transport system, small permease component

2. Cellular processes and signaling

Gene	Gene name	COG ID	Annotation
**COG category D: Cell cycle control, cell division, chromosome partitioning**			
atu2679		COG2755	Lysophospholipase L1 or related esterase. Includes spore coat protein LipC/YcsK
**COG category M: Cell wall/membrane/envelope biogenesis**			
atu0199		COG1732	Periplasmic glycine betaine/choline-binding (lipo)protein of an ABC-type transport system (osmoprotectant binding protein)
atu0661	*mfpsA*	COG0438	Lipopolysaccharide 1,6-galactosyltransferase, GT1 family
atu1406		COG4948	L-alanine-DL-glutamate epimerase or related enzyme of enolase superfamily
atu1877		COG2885	Outer membrane protein OmpA and related peptidoglycan-associated (lipo)proteins
atu2159	*omp*	COG3637	Opacity protein LomR and related surface antigens
atu5067	*dapA*	COG0329	4-hydroxy-tetrahydrodipicolinate synthase/N-acetylneuraminate lyase
atu5394	*dapA*	COG0329	4-hydroxy-tetrahydrodipicolinate synthase/N-acetylneuraminate lyase
**COG category O: Posttranslational modification, protein turnover, chaperones**			
atu0044	*hslV*	COG5405	ATP-dependent protease HslVU (ClpYQ), peptidase subunit
atu0045	*hslU*	COG1220	ATP-dependent protease HslVU (ClpYQ), ATPase subunit HslU
atu0375	*hspC*	COG0071	Small heat shock protein IbpA, HSP20 family
atu0637	*sohB*	COG0616	Periplasmic serine protease, ClpP class
atu0908	*msrB*	COG0229	Peptide methionine sulfoxide reductase MsrB
atu1121	*ligE*	COG0625	Glutathione S-transferase or stringent starvation protein SspA
atu2826	*htpX*	COG0501	Zn-dependent protease with chaperone function
atu3887	*hspL*	COG0071	Small heat shock protein IbpA, HSP20 family
atu4177	*clpB*	COG0542	ATP-dependent Clp protease, ATP-binding subunit ClpA
atu4764		COG1670	Protein N-acetyltransferase, RimJ/RimL family
atu5052	*hspAT1*	COG0071	Small heat shock protein IbpA, HSP20 family
atu5449	*hspAT2*	COG0071	Small heat shock protein IbpA, HSP20 family
atu8165		COG3785	Heat shock protein HspQ
**COG category T: Signal transduction mechanisms**			
atu2757	*glnK*	COG0347	Nitrogen regulatory protein PII
atu3357		COG0834	ABC-type amino acid transport/signal transduction system, periplasmic component/domain
atu4013	*cyaA-2*	COG2114	Adenylate cyclase, class 3
atu4162		COG0784	CheY-like REC (receiver) domain, includes chemotaxis protein CheY and sporulation regulator Spo0F

3. Information storage and processing

Gene	Gene name	COG ID	Annotation
**COG category J: Translation, ribosomal structure and biogenesis**			
atu4764		COG1670	Protein N-acetyltransferase, RimJ/RimL family
**COG category K: Transcription**			
atu1885		COG1522	DNA-binding transcriptional regulator, Lrp family
atu2021		COG2207	AraC-type DNA-binding domain and AraC-containing proteins
atu2445	*rpoH*	COG0568	DNA-directed RNA polymerase, sigma subunit (sigma70/sigma32)
atu4162		COG1595	DNA-directed RNA polymerase specialized sigma subunit, sigma24 family
atu4373	*cytR*	COG1609	DNA-binding transcriptional regulator, LacI/PurR family
atu5419		COG0583	DNA-binding transcriptional regulator, LysR family

4. Poorly characterized

Gene	Gene name	COG ID	Annotation
**COG category R: General function prediction only**			
atu0266		COG0673	Predicted dehydrogenase
atu0415		COG2070	NAD(P)H-dependent flavin oxidoreductase YrpB, nitropropane dioxygenase family
atu1250		COG1832	Predicted CoA-binding protein
atu1406		COG4948	L-alanine-DL-glutamate epimerase or related enzyme of enolase superfamily
atu2307		COG2141	Flavin-dependent oxidoreductase, luciferase family (includes alkanesulfonate monooxygenase SsuD and methylene tetrahydromethanopterin reductase)
atu2471	*tctA*	COG3333	TctA family transporter
atu2604		COG0673	Predicted dehydrogenase
atu3234		COG0673	Predicted dehydrogenase
atu3426	*ssuD*	COG2141	Flavin-dependent oxidoreductase, luciferase family (includes alkanesulfonate monooxygenase SsuD and methylene tetrahydromethanopterin reductase)
atu3428		COG3545	Predicted esterase of the alpha/beta hydrolase fold
atu3614	*gloB*	COG0491	Glyoxylase or a related metal-dependent hydrolase, beta-lactamase superfamily II
atu3726		COG2141	Flavin-dependent oxidoreductase, luciferase family (includes alkanesulfonate monooxygenase SsuD and methylene tetrahydromethanopterin reductase)
atu4012	*idhA*	COG0673	Predicted dehydrogenase
atu4377		COG2303	Choline dehydrogenase or related flavoprotein

Gene	Gene name	COG ID	Annotation
**COG category S: Function unknown**			
atu1009		COG4731	Uncharacterized conserved protein, DUF2147 family
atu2203		COG4991	Uncharacterized conserved protein YraI
atu2404		COG3791	Uncharacterized conserved protein
atu2655		COG4223	Uncharacterized conserved protein
atu3728		COG3871	General stress protein 26 (function unknown)
atu3752		COG5570	Uncharacterized conserved protein, DUF465 domain
atu4495		COG2979	Uncharacterized membrane protein YebE, DUF533 family

Downregulation: 40 DEGs

1. Metabolism

Gene	Gene name	COG ID	Annotation
**COG category C: Energy production and conversion**			
atu1530	*fixG*	COG0348	Polyferredoxin NapH
atu1654		COG0778	Nitroreductase
atu3298	*dctA*	COG1301	Na^+/H^+-dicarboxylate symporter
atu4391	*norE*	COG1845	Heme/copper-type cytochrome/quinol oxidase, subunit 3
**COG category E: Amino acid transport and metabolism**			
atu1437		COG0697	Amino acid export permease, drug/metabolite transporter (DMT) superfamily
atu2364		COG4215	ABC-type arginine transport system, permease component
atu2413		COG0559	Branched-chain amino acid ABC-type transport system, permease component
atu3459		COG0601	ABC-type dipeptide/oligopeptide/nickel transport system, permease component
**COG category G: Carbohydrate transport and metabolism**			
atu2492	*mtbA*	COG2814	Predicted arabinose efflux permease AraJ, MFS family
atu3129	*picA*	COG5434	Polygalacturonase
atu4447		COG1653	ABC-type glycerol-3-phosphate transport system, periplasmic component
atu4448		COG1175	ABC-type sugar transport system, permease component
atu4449		COG0395	ABC-type glycerol-3-phosphate transport system, permease component
atu4450		COG3839	ABC-type sugar transport system, ATPase component MalK
atu4451	*mtlK*	COG0246	Mannitol-1-phosphate/altronate dehydrogenases
**COG category H: Coenzyme transport and metabolism**			
atu4394		COG0043	3-polyprenyl-4-hydroxybenzoate decarboxylase
atu4396		COG3154	Ubiquinone biosynthesis accessory factor UbiT, lipid carrier SCP2 domain
**COG category I: Lipid transport and metabolism**			
atu1416		COG0318	O-succinylbenzoic acid-CoA ligase MenE or related acyl-CoA synthetase (AMP-forming)
atu3401		COG1012	Acyl-CoA reductase or other NAD-dependent aldehyde dehydrogenase
**COG category P: Inorganic ion transport and metabolism**			
atu2274		COG0053	Divalent metal cation (Fe/Co/Zn/Cd) efflux pump
atu3459		COG0601	ABC-type dipeptide/oligopeptide/nickel transport system, permease component
atu4402		COG0600	ABC-type nitrate/sulfonate/bicarbonate transport system, permease component

2. Cellular processes and signaling

Gene	Gene name	COG ID	Annotation
**COG category M: Cell wall/membrane/envelope biogenesis**			
atu3272	*pssN*	COG1596	Periplasmic protein Wza involved in polysaccharide export, contains SLBB domain of the beta-grasp fold
atu3302	*celD*	COG4783	Periplasmic chaperone/metalloprotease BepA/YfgC, contains M48 and TPR domains
atu3558		COG0438	Lipopolysaccharide 1,6-galactosyltransferase, GT1 family
atu4081		COG1794	Amino acid racemase YgeA
atu4298		COG0668	Small-conductance mechanosensitive channel
atu4816	*bme7*	COG0438	Lipopolysaccharide 1,6-galactosyltransferase, GT1 family
**COG category N: Cell mobility**			
atu3302	*celD*	COG5653	Acetyltransferase involved in cellulose biosynthesis, CelD/BcsL family
**COG category O: Posttranslational modification, protein turnover, chaperones**			
atu0138	*surf*	COG3346	Cytochrome oxidase assembly protein ShyY1
atu3302	*celD*	COG4783	Periplasmic chaperone/metalloprotease BepA/YfgC, contains M48 and TPR domains
atu4381	*nirV*	COG1262	Formylglycine-generating enzyme, required for sulfatase activity, contains SUMF1/FGE domain

3. Information storage and processing

Gene	Gene name	COG ID	Annotation
**COG category K: Transcription**			
atu0672		COG0583	DNA-binding transcriptional regulator, LysR family
atu3121	*cspA-4*	COG1278	Cold shock protein, CspA family
atu4299		COG1959	DNA-binding transcriptional regulator, IscR family
atu4584		COG1309	DNA-binding protein, AcrR family, includes nucleoid occlusion protein SlmA
atu5513	*cspA*	COG1278	Cold shock protein, CspA family
**COG category L: Replication, recombination and repair**			
atu3588	*alkA*	COG0122	3-methyladenine DNA glycosylase/8-oxoguanine DNA glycosylase

4. Poorly characterized

Gene	Gene name	COG ID	Annotation
**COG category R: General function prediction only**			
atu1437		COG0697	Amino acid export permease, drug/metabolite transporter (DMT) superfamily
atu8174		COG3907	Membrane-associated enzyme, PAP2 (acid phosphatase) superfamily
**COG category S: Function unknown**			
atu0671		COG2855	Uncharacterized membrane protein YeiH, induced by redox stress, UPF0324 family
**COG category X: Not match to the database**			
atu4604		COG2801	Transposase InsO of transposable element IS3, Rve (retroviral integrase) family
