# Supplementary material for: Characterization of adaptive evolution strains for the development of triclosan resistance in Agrobacterium tumefaciens C58
Source: Appl Environ Microbiol. 2026 Jan 6;92(1):e01232-25. doi: 10.1128/aem.01232-25 (PMC12838394; doi:10.1128/aem.01232-25)
Supplement: Data Set S2 — KEGG pathway enrichment analysis. [file aem.01232-25-s0002.pdf]

Data Set 2 KEGG pathway enrichment analysis. Differentially expressed genes (DEGs, fold change > 2 and *q* value < 0.001) between WT and HDR-12a including 146 upregulated DEGs and 40 downregulated DEGs, were analyzed separately using KOBAS-i with KEGG pathway annotation. Statistical significance (*p* value < 0.05) was determined using the hypergeometric test. Significantly enriched pathways of DEGs are in bold letters and highlighted on the gray background.

Upregulated DEGs

ID	Term	DEGs in pathway	Total genes in pathway	*p* value	Gene
**atu00920**	**Sulfur metabolism**	7	38	1.23E-04	*atu0312* (*cysK*), *atu0817* (*cysD-2*), *atu0818* (*cysH*), *atu0820*, *atu3426* (*ssuD*), *atu3504*, *atu4154*
**atu02010**	**ABC transporters**	21	333	2.19E-04	*atu0064* (*frcC*), *atu0065* (*frcA*), *atu0199*, *atu0591* (*aglE*), *atu0592* (*aglF*), *atu0593* (*aglG*), *atu0820*, *atu3063*, *atu3338* (*thuE*), *atu3504*, *atu3891*, *atu3893*, *atu4154*, *atu4320* (*rbsB-4*), *atu4369* (*rbsB-5*), *atu4370*, *atu4371*, *atu4468* (*sitD*), *atu4469* (*sitC*), *atu4470* (*sitB*), *atu4471* (*sitA*)
**atu00261**	**Monobactam biosynthesis**	3	15	1.00E-02	*atu0817* (*cysD-2*), *atu5067* (*dapA*), *atu5394* (*dapA*)
**atu00071**	**Fatty acid degradation**	3	24	3.04E-02	*atu0405* (*fadD*), *atu0502*, *atu0503* (*fadB-2*)
**atu00310**	**Lysine degradation**	3	25	3.35E-02	*atu0502*, *atu0503* (*fadB-2*), *atu3498*
atu00650	Butanoate metabolism	3	34	6.74E-02	*atu0502*, *atu0503* (*fadB-2*), *atu3498*
atu01212	Fatty acid metabolism	3	36	7.64E-02	*atu0405* (*fadD*), *atu0502*, *atu0503* (*fadB-2*)
atu00380	Tryptophan metabolism	2	21	1.16E-01	*atu0502*, *atu0503* (*fadB-2*)
atu00300	Lysine biosynthesis	2	21	1.16E-01	*atu5067* (*dapA*), *atu5394 (dapA)*
atu00760	Nicotinate and nicotinamide metabolism	2	23	1.33E-01	*atu3498*, *atu4097* (*nadB*)
atu02030	Bacterial chemotaxis	3	47	1.35E-01	*atu3063*, *atu4320* (*rbsB-4*), *atu4369* (*rbsB-5*)
atu00281	Geraniol degradation	1	5	1.44E-01	*atu0503* (*fadB-2*)
atu01120	Microbial metabolism in diverse environments	10	259	1.48E-01	*atu0312* (*cysK*), *atu0502*, *atu0503* (*fadB-2*), *atu0817* (*cysD-2*), *atu0818* (*cysH*), *atu1454* (*cysG*), *atu3498*, *atu4012* (*idhA*), *atu5067* (*dapA*), *atu5394* (*dapA*)
atu00362	Benzoate degradation	2	26	1.59E-01	*atu0502*, *atu0503* (*fadB-2*)

ID	Term	DEGs in pathway	Total genes in pathway	*p* value	Gene
atu00270	Cysteine and methionine metabolism	2	29	1.87E-01	*atu0312* (*cysK*), *atu1251* (*cysD*)
atu00072	Synthesis and degradation of ketone bodies	1	7	1.87E-01	*atu0502*
atu01130	Biosynthesis of antibiotics	9	242	1.91E-01	*atu0312* (*cysK*), *atu0502*, *atu0503* (*fadB-2*), *atu0817* (*cysD-2*), *atu4007* (*arcA*), *atu4012* (*idhA*), *atu4135* (*gcd-2*), *atu5067* (*dapA*), *atu5394* (*dapA*)
atu00640	Propanoate metabolism	2	30	1.96E-01	*atu0502*, *atu0503* (*fadB-2*)
atu00903	Limonene and pinene degradation	1	8	2.08E-01	*atu0503* (*fadB-2*)
atu00280	Valine, leucine and isoleucine degradation	2	33	2.24E-01	*atu0502*, *atu0503* (*fadB-2*)
atu00450	Selenocompound metabolism	1	10	2.48E-01	*atu0817* (*cysD-2*)
atu00250	Alanine, aspartate and glutamate metabolism	2	37	2.63E-01	*atu3498*, *atu4097 (nadB)*
atu00521	Streptomycin biosynthesis	1	13	3.04E-01	*atu4012* (*idhA*)
atu00900	Terpenoid backbone biosynthesis	1	13	3.04E-01	*atu0502*
atu00410	beta-Alanine metabolism	1	13	3.04E-01	*atu0503* (*fadB-2*)
atu00562	Inositol phosphate metabolism	1	14	3.22E-01	*atu4012* (*idhA*),
atu00620	Pyruvate metabolism	2	45	3.38E-01	*atu0502*, *atu3614 (gloB)*
atu02020	Two-component system	4	115	3.61E-01	*atu0502*, *atu2469* (*tctC*), *atu2470* (*tctB*), *atu2471* (*tctA*)
atu00350	Tyrosine metabolism	1	19	4.04E-01	*atu3498*
atu00910	Nitrogen metabolism	1	23	4.63E-01	*atu0415*
atu01230	Biosynthesis of amino acids	4	133	4.65E-01	*atu0312* (*cysK*), *atu4007* (*arcA*), *atu5067* (*dapA*), *atu5394* (*dapA*)
atu00220	Arginine biosynthesis	1	26	5.03E-01	*atu4007* (*arcA*)
atu00061	Fatty acid biosynthesis	1	27	5.16E-01	*atu0405* (*fadD*)
atu01110	Biosynthesis of secondary metabolites	8	297	5.21E-01	*atu0312* (*cysK*), *atu0502*, *atu0503* (*fadB-2*), *atu1454* (*cysG*), *atu4007* (*arcA*), *atu4135* (*gcd-2*), *atu5067* (*dapA*), *atu5394* (*dapA*)
atu00230	Purine metabolism	2	69	5.45E-01	*atu0817* (*cysD-2*), *atu4013* (*cyaA-2*)
atu01200	Carbon metabolism	3	109	5.49E-01	*atu0312* (*cysK*), *atu0502*, *atu0503* (*fadB-2*)
atu00030	Pentose phosphate pathway	1	31	5.64E-01	*atu4135* (*gcd-2*)
atu00330	Arginine and proline metabolism	1	34	5.97E-01	*atu4007* (*arcA*)
atu00860	Porphyrin and chlorophyll metabolism	1	39	6.46E-01	*atu1454* (*cysG*)
atu00630	Glyoxylate and dicarboxylate metabolism	1	60	7.95E-01	*atu0502*

Downregulated DEGs

ID	Term	DEGs in pathway	Total genes in pathway	*p* value	Gene
atu00903	Limonene and pinene degradation	1	8	6.06E-02	*atu3401*
atu00130	Ubiquinone and other terpenoid-quinone biosynthesis	1	9	6.71E-02	*atu4394*
atu00740	Riboflavin metabolism	1	10	7.35E-02	*atu1654*
atu02010	ABC transporters	5	333	8.10E-02	*atu2413, atu4447, atu4448, atu4449, atu4450*
atu00410	beta-Alanine metabolism	1	13	9.27E-02	*atu3401*
atu03410	Base excision repair	1	16	1.11E-01	*atu3588 (alkA)*
atu00561	Glycerolipid metabolism	1	16	1.11E-01	*atu3401*
atu00625	Chloroalkane and chloroalkene degradation	1	17	1.18E-01	*atu3401*
atu00053	Ascorbate and aldarate metabolism	1	18	1.24E-01	*atu3401*
atu00051	Fructose and mannose metabolism	1	20	1.36E-01	*atu4451 (mtlK)*
atu00380	Tryptophan metabolism	1	21	1.42E-01	*atu3401*
atu00340	Histidine metabolism	1	22	1.48E-01	*atu3401*
atu00071	Fatty acid degradation	1	24	1.60E-01	*atu3401*
atu00310	Lysine degradation	1	25	1.65E-01	*atu3401*
atu02020	Two-component system	2	115	1.93E-01	*atu3272* (*pssN*), *atu3298* (*dctA*)
atu00280	Valine, leucine and isoleucine degradation	1	33	2.11E-01	*atu3401*
atu00010	Glycolysis / Gluconeogenesis	1	34	2.16E-01	*atu3401*
atu00330	Arginine and proline metabolism	1	34	2.16E-01	*atu3401*
atu00250	Alanine, aspartate and glutamate metabolism	1	37	2.32E-01	*atu4081*
atu00620	Pyruvate metabolism	1	45	2.74E-01	*atu3401*
atu01110	Biosynthesis of secondary metabolites	2	297	6.21E-01	*atu3401, atu4394*
atu01100	Metabolic pathways	5	853	7.29E-01	*atu1654, atu3401, atu4081, atu4394, atu4451 (mtlK)*
atu02024	Quorum sensing	1	200	7.58E-01	*atu3459*
atu01130	Biosynthesis of antibiotics	1	242	8.22E-01	*atu3401*
atu01120	Microbial metabolism in diverse environments	1	259	8.42E-01	*atu3401*
