# Supplementary material for: Characterization of adaptive evolution strains for the development of triclosan resistance in Agrobacterium tumefaciens C58
Source: Appl Environ Microbiol. 2026 Jan 6;92(1):e01232-25. doi: 10.1128/aem.01232-25 (PMC12838394; doi:10.1128/aem.01232-25)
Supplement: Data Set S3 — PPI network analysis. [file aem.01232-25-s0003.pdf]

Data Set 3 Protein-protein interaction (PPI) network analysis of 146 upregulated DEGs of HDR-12a generating via STRING version 12.0 with the medium confidence score 0.40. Numbers inside each node indicate fold induction from transcriptomic analysis.

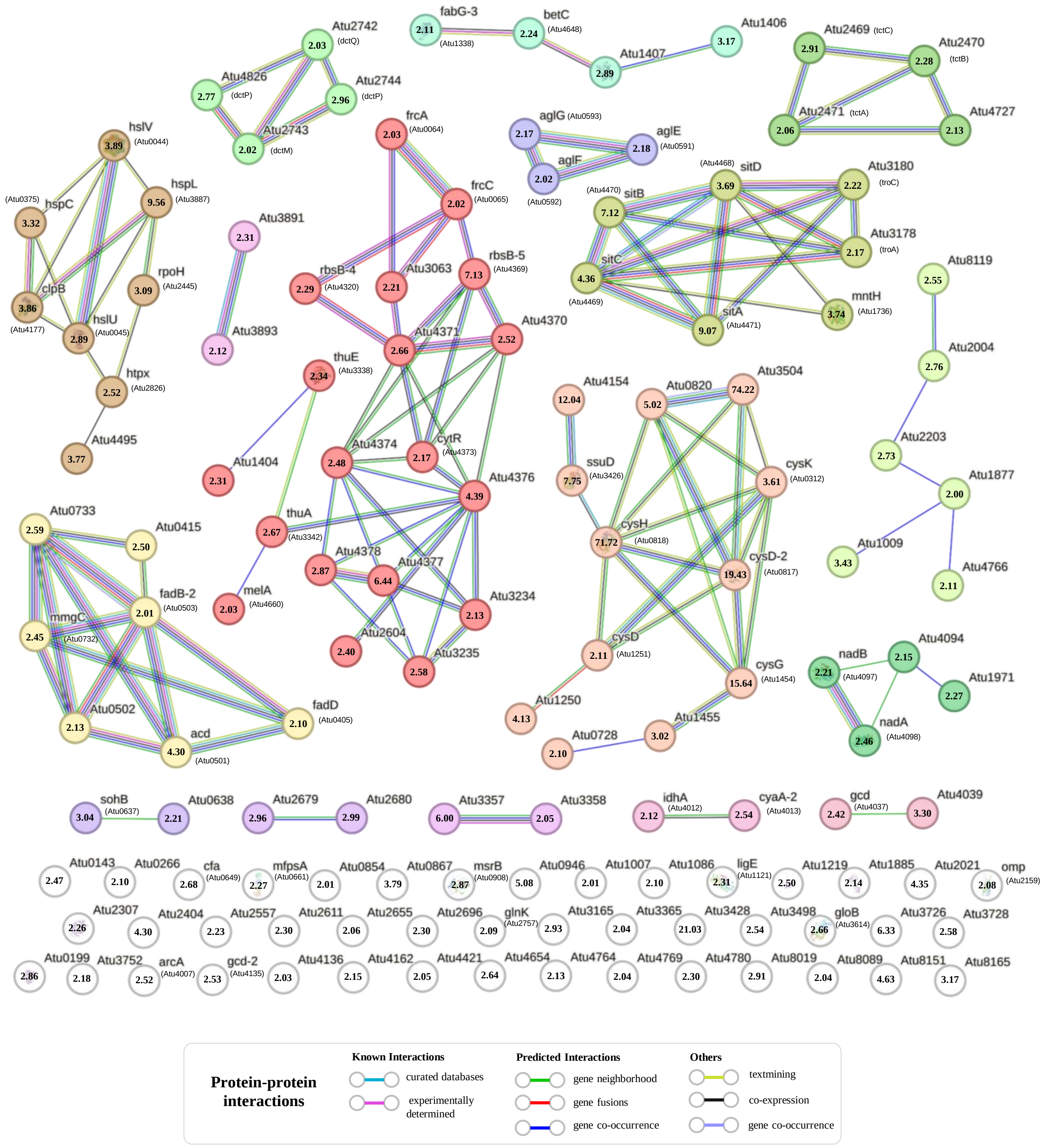

k-means clustering of the upregulated DEGs

Color	Cluster ID	Count	Description	Protein name
	Cluster 1	19	Gfo/Idh/MocA-like oxidoreductase, C-terminal, and Xylose isomerase-like TIM barrel	Atu4370, Atu4371, cytR (Atu4373), Atu4374, Atu4377, Atu4378, Atu4376, thuA (Atu3342), melA (Atu4660), thuE (Atu3338), Atu1404, Atu3235, Atu3234, Atu2604, rbsB-5 (Atu4369), frcC (Atu0064), frcA (Atu0065), Atu3063, rbsB-4 (Atu4320)
	Cluster 2	12	Sulfur metabolism	cysD (Atu1251), Atu1250, cysH (Atu0818), cysG (Atu1454), Atu1455, Atu0728, Atu0820, Atu3504, cysD-2 (Atu0817), cysK (Atu0312), ssuD (Atu3426), Atu4154
	Cluster 3	8	I. Stress response<br>II. Protein folding, and ATP-dependent peptidase activity	hspC (Atu0375), clpB (Atu4177), hspL (Atu3887), rpoH (Atu2445), htpx (Atu2826), Atu4495, hslU (Atu0045), hslV (Atu0044)
	Cluster 4	7	Valine, leucine and isoleucine degradation, and Acyl-CoA dehydrogenase/oxidase C-terminal	mmgC (Atu0732), Atu0733, fadB-2 (Atu0503), Atu0502, acd (Atu0501), fadD (Atu0405), Atu0415
	Cluster 5	7	ABC transporter, TroCD-like, and Adhesin B	sitB (Atu4470), sitA (Atu4471), sitC (Atu4469), sitD (Atu4468), Atu3180 (troC), Atu3178 (troA), mntH (Atu1736)
	Cluster 6	6	I. Sporulation resulting in formation of a cellular spore<br>II. Mostly uncharacterized, incl. PRC-barrel domain, and Uracil-DNA glycosylase family 4	Atu4766, Atu1877, Atu2203, Atu2004, Atu8119, Atu1009
	Cluster 7	4	Protein of unknown function DUF1468, and Protein of unknown function DUF112, transmembrane	Atu2470 (tctB), Atu2471 (tctA), Atu4727 (tctC), Atu2469 (tctC)
	Cluster 8	4	TRAP transporter solute receptor, DctP family, and TRAP transporter large membrane protein DctM	Atu2743 (dctM), Atu2744 (dctP), Atu2742 (dctQ), Atu4826 (dctP)
	Cluster 9	4	Pyridine nucleotide biosynthesis	nadA (Atu4098), nadB (Atu4097), Atu4094, Atu1971
	Cluster 10	4	Mixed, incl. L-threonine catabolism to glycine, and D-galactonate dehydratase DgoD-like	betC (Atu4648), Atu1407, Atu1406, fabG-3 (Atu1338)
	Cluster 11	3	Mixed, incl. Periplasmic binding protein-like domain, and Bacterial extracellular solute-binding protein	aglE (Atu0591), aglF (Atu0592), aglG (Atu0593)
	Cluster 12	2	Mixed, incl. Peptidase S49, and Protein ubiquitination	sohB (Atu0637), Atu0638
	Cluster 13	2	Mixed, incl. RNA ligase activity, and aspartate-tRNA ligase activity	Atu2679, Atu2680
	Cluster 14	2	Ligand-gated channel activity, and ABC transporter membrane protein permease protein ArtM/GltK/GlnP/TcyL/YhdX-like	Atu3358, Atu3357
	Cluster 15	2	ABC transporter, ferric cation import, FbpC, and Bacterial extracellular solute-binding protein	Atu3893, Atu3891
	Cluster 16	2	-	cyaA-2 (Atu4013), idhA (Atu4012)
	Cluster 17	2	Mostly uncharacterized, incl. MAPEG family, and Glycolytic fermentation	gcd (Atu4037), Atu4039

Data Set 3 Protein-protein interaction (PPI) network analysis of 40 downregulated DEGs of HDR-12a generating via STRING version 12.0 with the medium confidence score 0.40. Numbers inside each node indicate fold reduction from transcriptomic analysis.

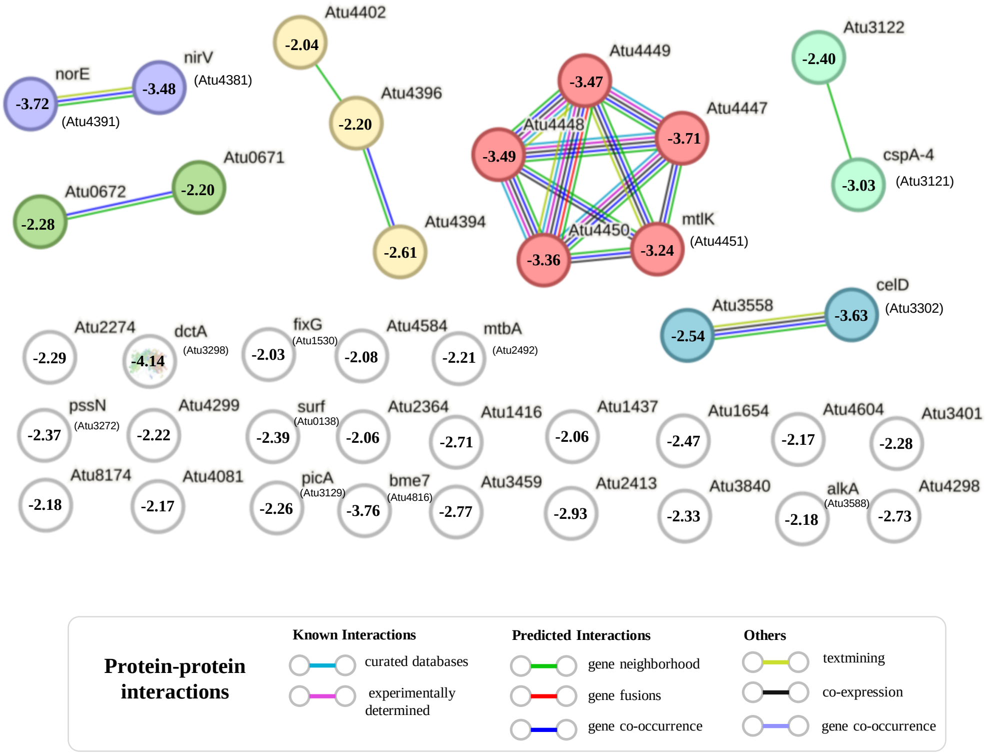

k-means clustering of the downregulated DEGs.

Color	Cluster ID	Count	Description	Protein name
	Cluster 1	5	Mixed, incl. TOBE domain, and ABC-type carbohydrate transporter activity	Atu4447, Atu4448, Atu4449, Atu4450, mtlK (Atu4451)
	Cluster 2	3	Mixed, incl. Quinone metabolism, and Nitrogen metabolism	Atu4394, Atu4396, Atu4402
	Cluster 3	2	-	Atu0671, Atu0672
	Cluster 4	2	-	cspA-4 (Atu3121), Atu3122
	Cluster 5	2	-	celD (Atu3302), Atu3558
	Cluster 6	2	-	norE (Atu4391), nirV (Atu4381)
