# Supplementary material for: Characterization of adaptive evolution strains for the development of triclosan resistance in Agrobacterium tumefaciens C58
Source: Appl Environ Microbiol. 2026 Jan 6;92(1):e01232-25. doi: 10.1128/aem.01232-25 (PMC12838394; doi:10.1128/aem.01232-25)
Supplement: Supplemental figures — Figures S1 to S6. [file aem.01232-25-s0004.pdf]

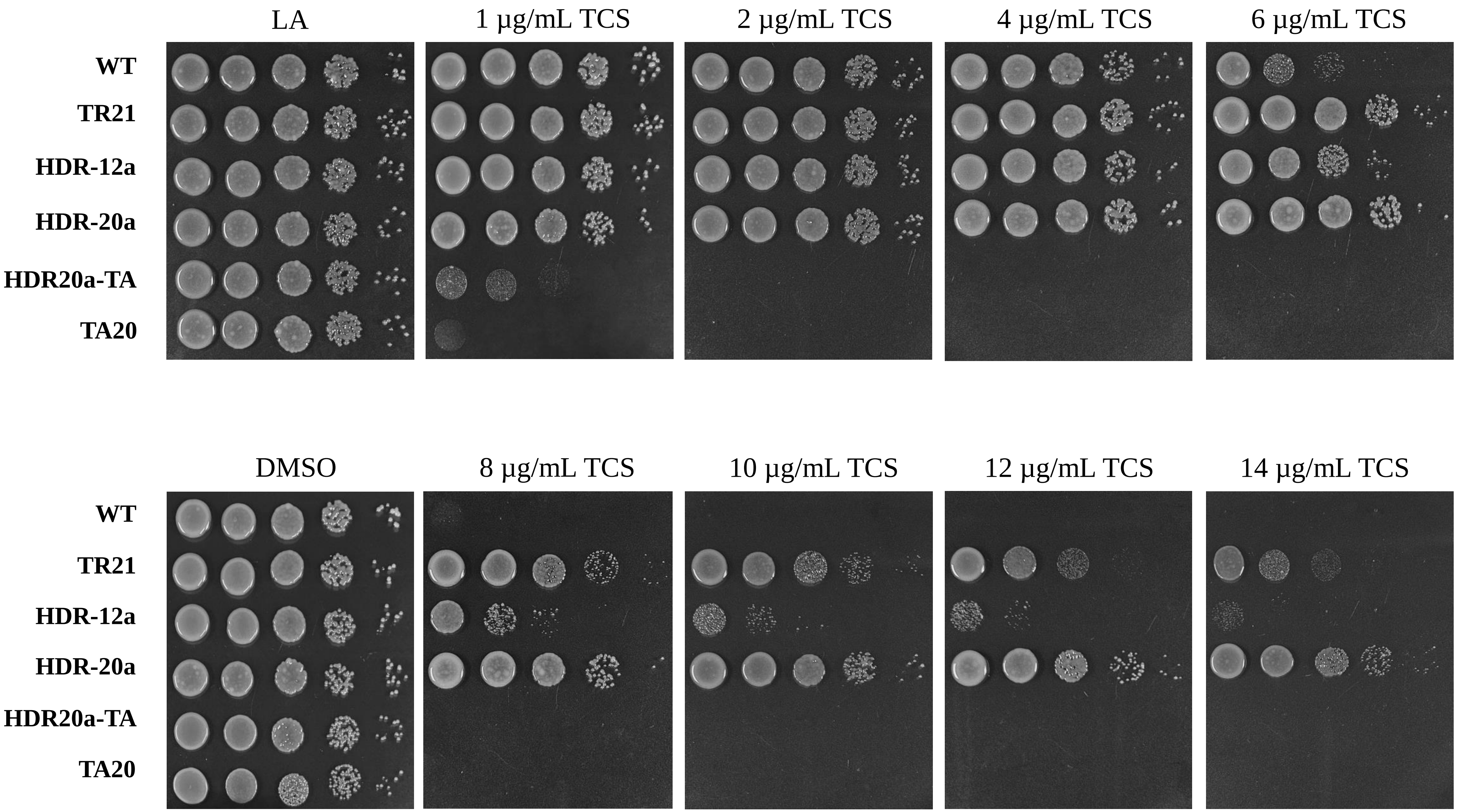
LA
1 µg/mL TCS
2 µg/mL TCS
4 µg/mL TCS
6 µg/mL TCS
WT
TR21
HDR-12a
HDR-20a
HDR20a-TA
TA20
DMSO
8 µg/mL TCS
10 µg/mL TCS
12 µg/mL TCS
14 µg/mL TCS
WT
TR21
HDR-12a
HDR-20a
HDR20a-TA
TA20

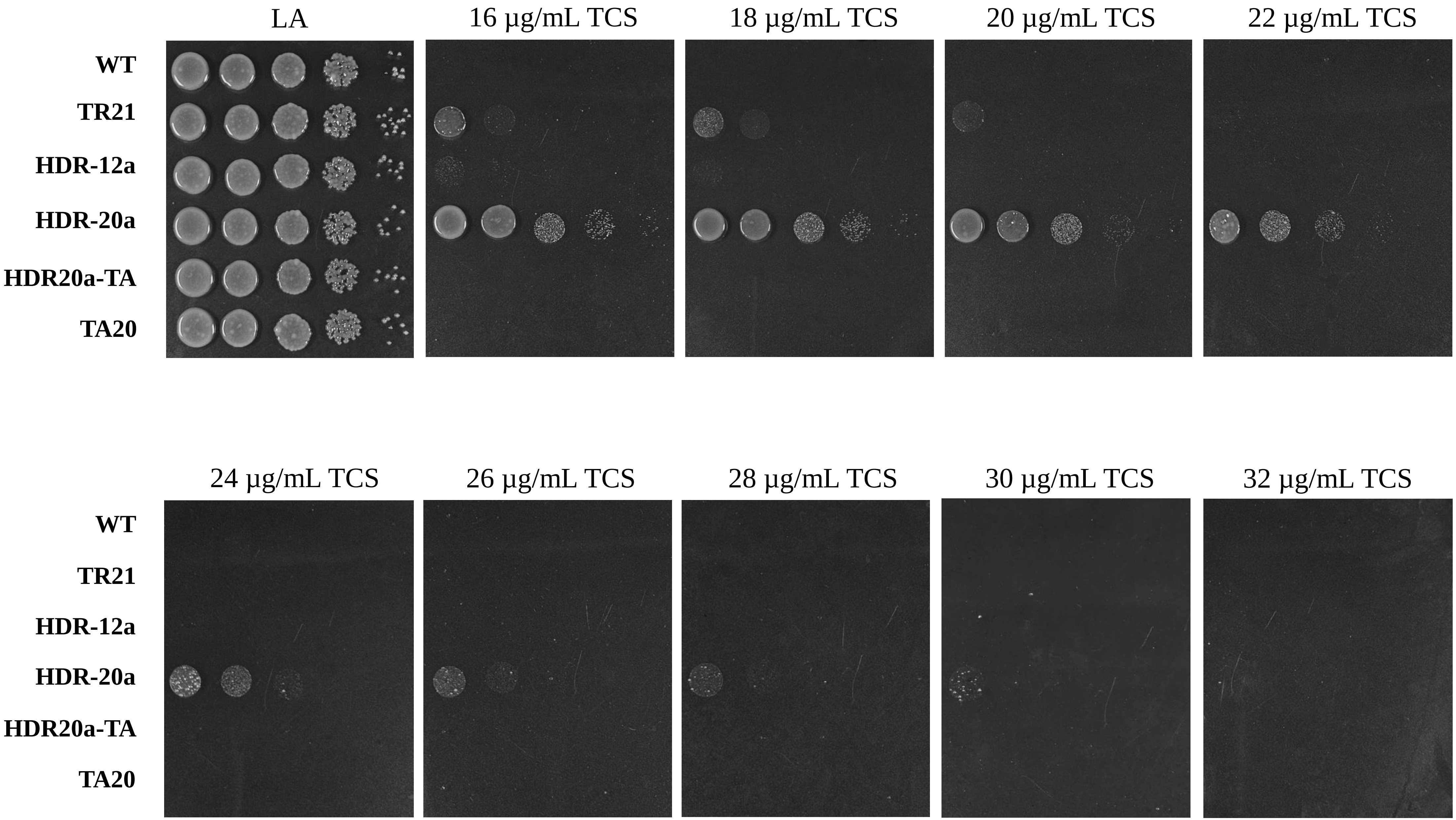
LA
16 μg/mL TCS
18 μg/mL TCS
20 μg/mL TCS
22 μg/mL TCS
WT
TR21
HDR-12a
HDR-20a
HDR20a-TA
TA20
24 μg/mL TCS
26 μg/mL TCS
28 μg/mL TCS
30 μg/mL TCS
32 μg/mL TCS
WT
TR21
HDR-12a
HDR-20a
HDR20a-TA
TA20

FIG S1 Plate sensitivity assay and determination of minimum inhibitory concentration (MIC). *A. tumefaciens* strains were WT (wild-type NTL4), TR21 (*triR* inactivation), HDR-12a, HDR-20a, HDR20a-TA (HDR-20a with *triA* disruption), TA20 (*triA* inactivation). Exponential growth phase cells grown in LB were adjusted in LB to yield an optical density of 600 nm (OD_{600}) of 0.125. Ten-fold serial dilutions were made in LB, and 10 µl of each dilution was spotted on LA or LA-containing various concentrations of TCS (1–32 µg/mL in increments of 2). TCS was dissolved in DMSO. An LA plate containing an equal amount of DMSO was used as a control. The plates were then incubated at 28°C for 48 h. The MIC was defined as the lowest concentration of TCS that inhibited visible growth of bacteria.

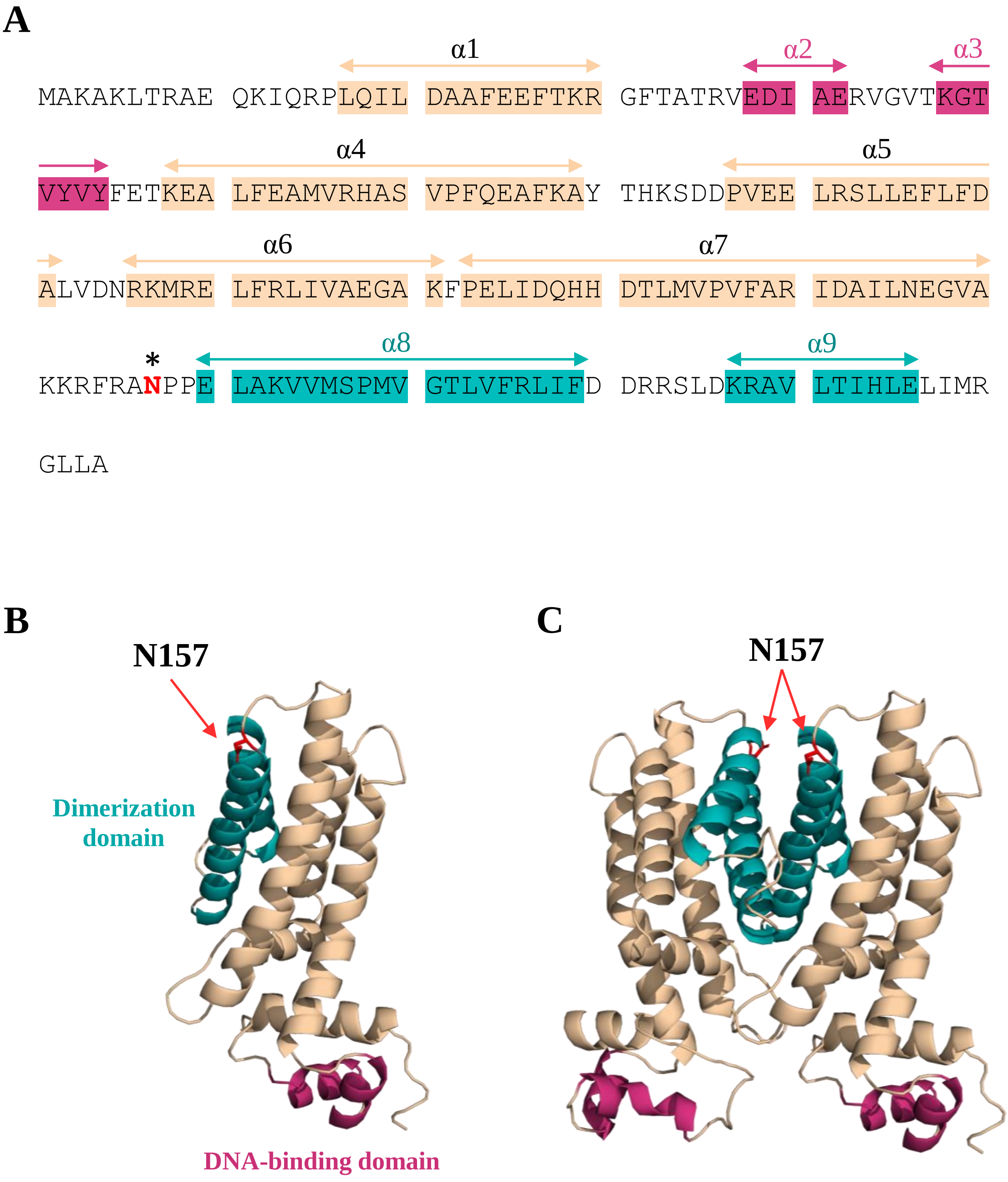

FIG S2 (A) Amino acid sequence and secondary structure of *A. tumefaciens* TriR. The Asn residue 157 (N157) is shown in red bold letter and marked with an asterisk. Structural models of TriR monomer (B) and homodimer (C). The crystal structure of *Stenotrophomonas maltophilia* SmeT (PDB ID: 3P9T) was used as the template for generating the structural model of TriR using the SWISS-MODEL, and PyMOL was used for visualization. The HTH DNA-binding motifs (helices 2 and 3) and predicted dimer interface (helices 8 and 9) are shown in pink and green, respectively. Helices 5 to 7 (brown) form a central triangle. The N157 is shown in red stick and marked with arrows.

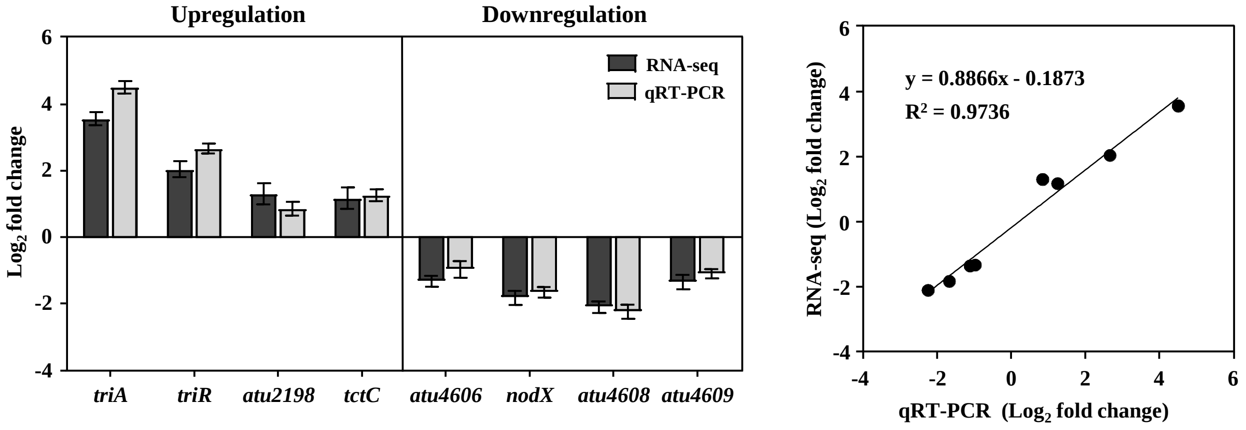

FIG S3 Validation of RNA-seq data using qRT-PCR. The upregulated and downregulated DEGs of HDR-20a were subjected to analysis. Black bars represents the RNA-seq results and gray bars represents the qRT-PCR results. R^2 indicates the linear coefficient of determination between the RNA-seq and qRT-PCR results.

A

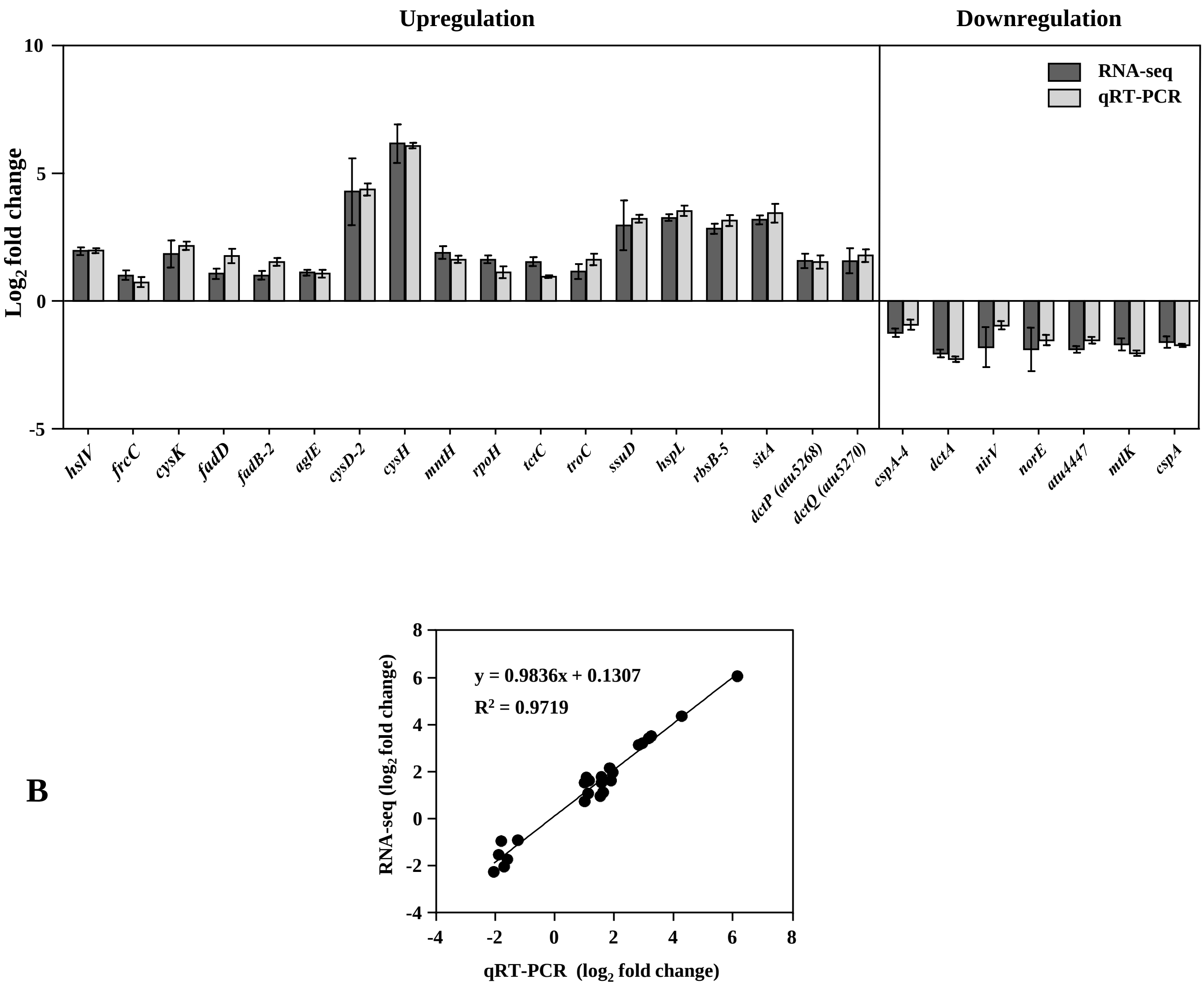

FIG S4 Validation of RNA-seq data using qRT-PCR. Selected upregulated and downregulated DEGs of HDR-12a were subjected to analysis. Black bars represents the RNA-seq results and gray bars represents the qRT-PCR results. R^2 indicates the linear coefficient of determination between the RNA-seq and qRT-PCR results.

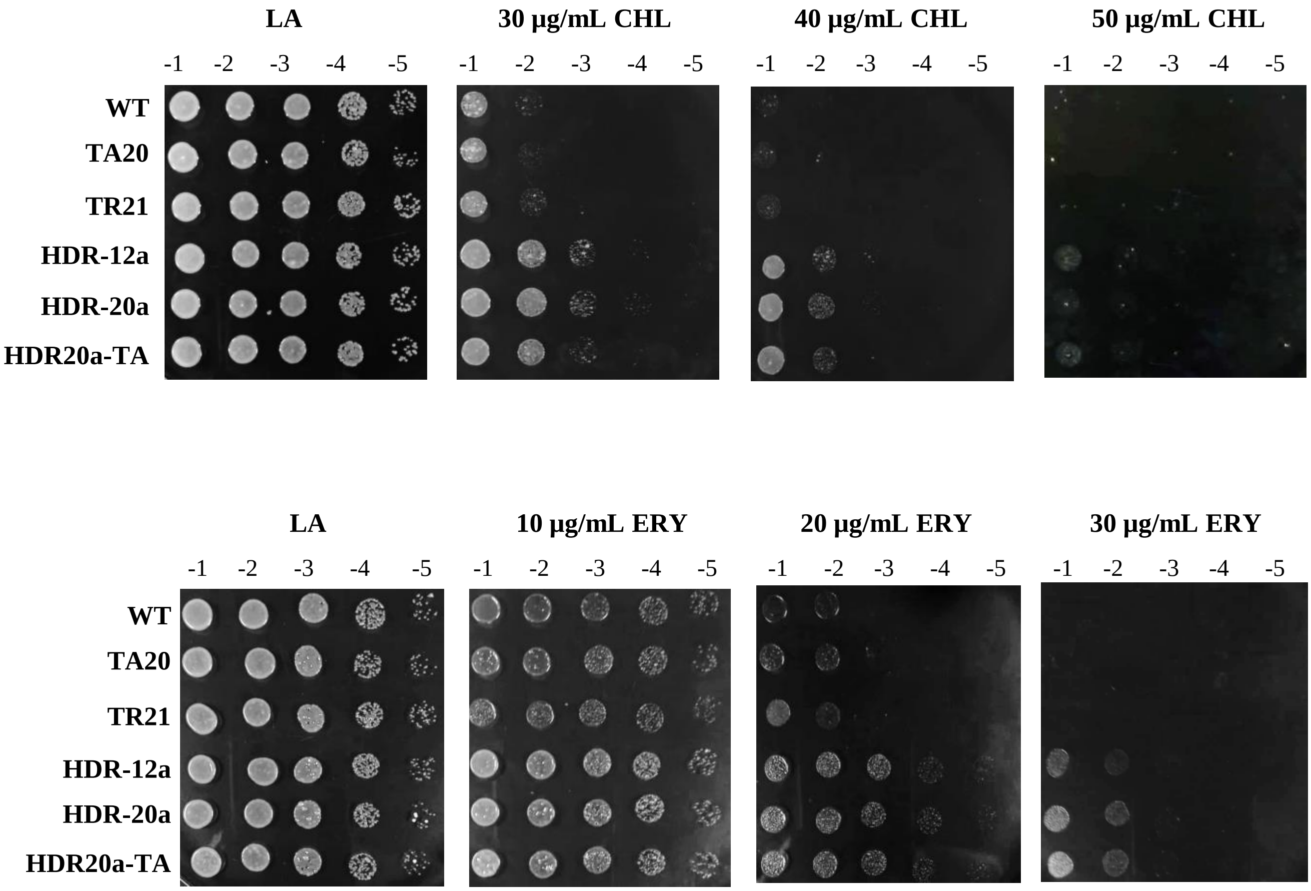

FIG S5 Plate sensitivity assay. Exponential phase cells of WT, TR20 (*triA* disruption), TR21 (*triR* disruption), HDR-12a, HDR-20a and HDR20a-TA (HDR-20a with *triA* disruption) were adjusted, 10-fold serial diluted and grown on LB agar (LA) plates containing chloramphenicol (30, 40 and 50 µg/mL CHL) and erythromycin (10, 20 and 30 µg/mL ERY). Plates were incubated at 28°C for 48 h.

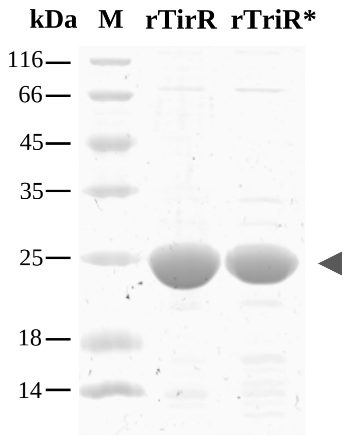

FIG S6 Purified recombinant proteins, rTriR and rTriR* (with a short peptide consisting of eight amino acids, WSHPQFEK, fused in-frame to the C-terminus). rTriR and rTriR* are wild-type TriR and mutated TriR (Asn157Thr) respectively. Fifteen µg of total protein was separated via 12.5% SDS-PAGE. The gel was stained with Coomassie blue. M: molecular weight protein ladders. The calculated molecular mass of rTriR is 24.6 kDa as indicated by a triangle.
