# Supplementary material for: Characterization of adaptive evolution strains for the development of triclosan resistance in Agrobacterium tumefaciens C58
Source: Appl Environ Microbiol. 2026 Jan 6;92(1):e01232-25. doi: 10.1128/aem.01232-25 (PMC12838394; doi:10.1128/aem.01232-25)
Supplement: Supplemental tables — Tables S1 and S2. [file aem.01232-25-s0005.pdf]

TABLE S1 List of differentially expressed genes (DEGs) of WT versus HDR-12a.
Log_2 fold change >1 or <-1 (a fold change greater than two) and q <0.001 were used as a threshold.

Upregulation: 146 genes

Gene locus	Gene Name	Description	Fold change	*q* value
atu3504		sulfate/thiosulfate transport system substrate-binding protein	74.22	3.64E-45
atu0818	*cysH*	phosphoadenosine phosphosulfate reductase	71.72	2.30E-42
atu3428		serine hydrolase	21.03	3.87E-06
atu0817	*cysD-2*	sulfate adenylyltransferase subunit 2	19.43	1.47E-07
atu1454	*cysG*	uroporphyrin-III C-methyltransferase / precorrin-2 dehydrogenase / sirohydrochlorin ferrochelatase	15.64	2.39E-43
atu4154		sulfonate transport system substrate-binding protein	12.04	1.85E-06
atu3887	*hspL*	small heat shock protein	9.56	0.00E+00
atu4471	*sitA*	manganese/iron transport system substrate-binding protein	9.07	3.54E-239
atu3426	*ssuD*	alkanesulfonate monooxygenase	7.75	1.30E-06
atu4369	*rbsB-5*	ribose transport system substrate-binding protein	7.13	3.63E-133
atu4470	*sitB*	manganese/iron transport system ATP-binding protein	7.12	1.96E-118
atu4377		glucoside 3-dehydrogenase (cytochrome c) catalytic subunit [oxidoreductase]	6.44	9.10E-98
atu3726		flavin dependant oxidoreductase	6.33	2.79E-200
atu3357		polar amino acid transport system substrate-binding protein	6.00	6.34E-12
atu0946		dehydrogenase	5.08	5.09E-239
atu0820		sulfate/thiosulfate transport system substrate-binding protein	5.02	2.57E-08
atu8151		hypothetical protein	4.63	1.27E-17
atu5052	*hspAT1*	molecular chaperone IbpA	4.47	5.66E-24
atu4376		conserved hypothetical protein	4.39	6.27E-78
atu4469	*sitC*	manganese/iron transport system permease protein	4.36	1.11E-38
atu2021		transcriptional regulator, AraC family	4.35	5.95E-71
atu0501	*acd*	3-(methylsulfanyl)propanoyl-CoA dehydrogenase	4.30	3.15E-117
atu2404		conserved hypothetical protein	4.30	7.53E-06
atu5419		transcriptional regulator, LysR family	4.20	5.08E-07
atu1250		uncharacterized protein	4.13	4.64E-56
atu5450		conserved hypothetical protein	3.89	9.13E-12
atu0044	*hslV*	ATP-dependent HslUV protease, peptidase subunit HslV	3.89	1.33E-109
atu4177	*clpB*	ATP-dependent Clp protease ATP-binding subunit ClpB	3.86	1.58E-227
atu0867		2,5-diketo-D-gluconate reductase B	3.79	5.82E-76

Gene locus	Gene name	Description	Fold change	*q* value
atu4495		conserved hypothetical protein	3.77	2.79E-141
atu1736	*mntH*	manganese transport protein	3.74	1.29E-37
atu4468	*sitD*	manganese/iron transport system permease protein	3.69	6.05E-18
atu0312	*cysK*	cysteine synthase	3.61	2.20E-08
atu5431		hypothetical protein	3.47	1.04E-22
atu1009		conserved hypothetical protein	3.43	3.65E-24
atu5449	*hspAT2*	heat-shock protein	3.34	1.64E-45
atu0375	*hspC*	small heat shock protein	3.32	2.04E-29
atu4039		hypothetical protein	3.30	6.86E-04
atu1406		isomerase/lactonizing enzyme	3.17	3.34E-21
atu8165		heat shock protein HspQ	3.17	4.09E-97
atu2445	*rpoH*	RNA polymerase sigma-32 factor	3.09	1.06E-78
atu0637	*sohB*	proteinase sohB	3.04	1.54E-70
atu1455		conserved hypothetical protein	3.02	2.49E-06
atu5268	*dctP*	TRAP-type transport, substrate binding protein	3.00	1.63E-21
atu2680		putative ABC transport system ATP-binding protein	2.99	1.19E-33
atu5270	*dctQ*	permease component of C4 dicarboxylate transporter	2.97	2.58E-07
atu2679		acyl-CoA thioesterase I	2.96	5.67E-33
atu2744	*dctP*	TRAP-type transport system large permease protein	2.96	2.59E-94
atu3165		polyol transport system substrate-binding protein	2.93	2.67E-11
atu2469	*tctC*	putative tricarboxylic transport membrane protein	2.91	1.26E-53
atu8019		conserved hypothetical protein	2.91	2.23E-19
atu1407		gluconate 5-dehydrogenase	2.89	4.92E-10
atu0045	*hslU*	ATP-dependent HslUV protease ATP-binding subunit HslU	2.89	1.11E-85
atu4378		glucoside 3-dehydrogenase (cytochrome c) hitch-hiker subunit	2.87	5.24E-14
atu0908	*msrB*	peptide-methionine (R)-S-oxide reductase	2.87	2.89E-62
atu0199		osmoprotectant transport system substrate-binding protein	2.86	4.46E-35
atu4826	*dctP*	TRAP-type transport system periplasmic protein	2.77	2.30E-06
atu2004		conserved hypothetical protein	2.76	1.18E-42
atu2203		conserved hypothetical protein	2.73	6.24E-13
atu0649	*cfa*	cyclopropane-fatty-acyl-phospholipid synthase	2.68	9.10E-39
atu3342	*thuA*	trehalose utilization-related protein	2.67	1.07E-29
atu4371		ribose transport system permease protein	2.66	2.88E-07
atu3614	*gloB*	hydroxyacylglutathione hydrolase	2.66	9.87E-73

Gene locus	Gene name	Description	Fold change	*q* value
atu4654		multiple sugar transport system substrate-binding protein	2.64	3.90E-42
atu0733		enoyl-CoA hydratase/isomerase	2.59	4.51E-07
atu3235		conserved hypothetical protein	2.58	5.52E-23
atu3728		conserved hypothetical protein	2.58	6.35E-06
atu8119		conserved hypothetical protein	2.55	6.96E-05
atu4013	*cyaA-2*	adenylate cyclase	2.54	3.56E-51
atu3498		succinate-semialdehyde dehydrogenase / glutarate-semialdehyde dehydrogenase	2.54	3.94E-77
atu4135	*gcd-2*	quinoprotein glucose dehydrogenase	2.53	3.59E-48
atu4007	*arcA*	arginase	2.52	1.22E-28
atu2826	*htpX*	heat shock protein HtpX	2.52	2.01E-29
atu4370		ribose transport system ATP-binding protein	2.52	1.39E-08
atu1219		conserved hypothetical protein	2.50	8.81E-33
atu0415		nitronate monooxygenase	2.50	2.14E-23
atu4374		conserved hypothetical protein	2.48	4.19E-19
atu0143		MFS permease	2.47	3.71E-12
atu4098	*nadA*	quinolinate synthase	2.46	6.48E-28
atu0732	*mmgC*	3-(methylsulfanyl)propanoyl-CoA dehydrogenase	2.45	2.64E-28
atu5494		conserved hypothetical protein	2.45	2.78E-07
atu4037	*gcd*	aldose sugar dehydrogenase	2.42	5.18E-35
atu2604		oxidoreductase	2.40	8.89E-18
atu5071	*dppA*	peptide/nickel transport system substrate-binding protein	2.36	1.76E-22
atu3338	*thuE*	trehalose/maltose transport system substrate-binding protein	2.34	1.69E-12
atu1121	*ligE*	lignin degradation protein	2.31	4.69E-32
atu1404		multiple sugar transport system substrate-binding protein	2.31	1.79E-04
atu3891		glycerol transport system substrate-binding protein	2.31	1.60E-15
atu4780		conserved hypothetical protein	2.30	8.42E-15
atu2611		conserved hypothetical protein	2.30	2.54E-09
atu2696		conserved hypothetical protein	2.30	1.11E-32
atu4320	*rbsB-4*	ribose transport system substrate-binding protein	2.29	7.63E-32
atu2470	*tctB*	putative tricarboxylic transport membrane protein	2.28	2.88E-07
atu5394	*dapA*	4-hydroxy-tetrahydrodipicolinate synthase	2.28	2.83E-18
atu1971		conserved hypothetical protein	2.27	1.91E-31
atu0661	*mfpsA*	glycosyltransferase	2.27	4.78E-39
atu2307		monooxygenase	2.26	3.24E-22

Gene locus	Gene name	Description	Fold change	*q* value
atu5269	*dctM*	permease component of C4 dicarboxylate transporter	2.24	1.88E-11
atu4648	*betC*	choline-sulfatase	2.24	2.60E-14
atu2557		hypothetical protein	2.23	6.83E-09
atu3180	*troC*	zinc/manganese transport system ATP-binding protein	2.22	2.03E-10
atu3063		ribose transport system substrate-binding protein	2.21	5.03E-15
atu4097	*nadB*	L-aspartate oxidase	2.21	7.56E-13
atu0638		conserved hypothetical protein	2.21	1.34E-06
atu3752		conserved hypothetical protein	2.18	3.52E-20
atu0591	*aglE*	alpha-glucoside transport system substrate-binding protein	2.18	7.13E-68
atu4373	*cytR*	LacI family transcriptional regulator, repressor for deo operon, udp, cdd, tsx, nupC, and nupG	2.17	6.84E-11
atu3178	*troA*	zinc/manganese transport system substrate-binding protein	2.17	9.33E-10
atu0593	*aglG*	alpha-glucoside transport system permease protein	2.17	2.81E-59
atu4162		two component response regulator	2.15	1.04E-47
atu4094		hypothetical protein	2.15	6.97E-11
atu5067	*dapA*	4-hydroxy-tetrahydrodipicolinate synthase	2.14	1.03E-08
atu1885		Lrp/AsnC family transcriptional regulator, cysteine-sensing transcriptional activator	2.14	1.60E-04
atu0502		acetyl-CoA C-acetyltransferase	2.13	1.25E-14
atu4727		conserved hypothetical protein, TctC family	2.13	6.23E-13
atu4764		acetyltransferase	2.13	3.69E-12
atu3234		NAD binding oxidoreductase	2.13	2.05E-16
atu4012	*idhA*	myo-inositol 2-dehydrogenase / D-chiro-inositol 1-dehydrogenase	2.12	2.27E-19
atu3893		glycerol transport system permease protein	2.12	8.96E-05
atu1338	*fabG-3*	3-oxoacyl- (acyl carrier protein) reductase	2.11	2.80E-25
atu1251	*cysD*	O-acetylhomoserine (thiol)-lyase	2.11	2.79E-25
atu4766		hypothetical protein	2.11	8.39E-10
atu0728		conserved hypothetical protein	2.10	1.01E-12
atu0405	*fadD*	long-chain acyl-CoA synthetase	2.10	6.35E-18
atu0266		oxidoreductase	2.10	4.55E-20
atu1086		MFS permease	2.10	7.25E-09
atu2757	*glnK*	nitrogen regulatory protein P-II 2	2.09	1.40E-04
atu2159	*omp*	outer membrane immunogenic protein	2.08	4.26E-49
atu2655		conserved hypothetical protein	2.06	7.26E-50
atu2471	*tctA*	putative tricarboxylic transport membrane protein	2.06	2.20E-18

Gene locus	Gene name	Description	Fold change	*q* value
atu4421		putative spermidine/putrescine transport system substrate-binding protein	2.05	1.63E-35
atu3358		polar amino acid transport system permease protein	2.05	6.63E-09
atu4769		conserved hypothetical protein	2.04	5.86E-17
atu8089		conserved hypothetical protein	2.04	1.84E-04
atu3365		hypothetical protein	2.04	3.81E-12
atu4136		conserved hypothetical protein	2.03	1.42E-04
atu0065	*frcA*	fructose transport system ATP-binding protein	2.03	3.44E-19
atu2742	*dctQ*	TRAP-type transport system small permease protein	2.03	5.95E-10
atu4660	*melA*	alpha-galactosidase	2.03	6.91E-07
atu2743	*dctM*	TRAP-type transport system large permease protein	2.02	7.06E-31
atu0592	*aglF*	alpha-glucoside transport system permease protein	2.02	1.44E-47
atu0064	*frcC*	fructose transport system permease protein	2.02	1.11E-19
atu0503	*fadB-2*	3-hydroxyacyl-CoA dehydrogenase / enoyl-CoA hydratase / 3-hydroxybutyryl-CoA epimerase	2.01	5.44E-22
atu0854		conserved hypothetical protein	2.01	2.69E-08
atu1007		hypothetical protein	2.01	5.28E-05
atu1877		OmpA family protein	2.00	1.36E-52

Downregulation: 40 genes

Gene locus	Gene name	Description	Fold change	*q* value
atu3298	*dctA*	aerobic C4-dicarboxylate transport protein	-4.14	3.11E-120
atu4816	*bme7*	glycosyltransferase	-3.76	5.08E-05
atu4391	*norE*	nitric oxide reductase NorE protein	-3.72	7.10E-04
atu4447		polyol transport system substrate-binding protein	-3.71	9.24E-120
atu3302	*celD*	cellulose biosynthesis protein	-3.63	1.49E-05
atu4448		polyol transport system permease protein	-3.49	1.18E-13
atu4381	*nirV*	nitrite reductase, NirV precursor	-3.48	4.44E-04
atu4449		polyol transport system permease protein	-3.47	5.25E-15
atu4450		multiple sugar transport system ATP-binding protein	-3.36	1.18E-25
atu4451	*mtlK*	mannitol 2-dehydrogenase	-3.24	8.33E-34
atu5513	*cspA*	cold shock protein	-3.03	4.23E-34
atu2413		urea transport system permease protein	-2.93	3.64E-05
atu3459		peptide/nickel transport system permease protein	-2.77	6.34E-07
atu4298		moderate conductance mechanosensitive channel	-2.73	2.13E-04
atu1416		feruloyl-CoA synthase	-2.71	1.80E-05
atu4394		4-hydroxy-3-polyprenylbenzoate decarboxylase	-2.61	1.46E-06
atu3558		glycosyltransferase	-2.54	4.02E-04
atu1654		5,6-dimethylbenzimidazole synthase	-2.47	1.07E-13
atu3122		cold shock protein	-2.40	7.83E-55
atu0138	*surf*	surfeit locus 1 family protein	-2.39	1.26E-37
atu3121	*cspA-4*	cold shock protein	-2.37	7.93E-41
atu3272	*pssN*	polysaccharide biosynthesis/export protein	-2.37	3.68E-13
atu3840		hypothetical protein	-2.33	8.79E-04
atu2274		cation efflux system protein	-2.29	1.79E-32
atu0672		transcriptional regulator, LysR family	-2.28	1.56E-13
atu3401		aldehyde dehydrogenase (NAD+)	-2.28	7.44E-04
atu3129	*picA*	polygalacturonase-like protein	-2.26	5.56E-04
atu4299		conserved hypothetical protein	-2.22	4.38E-12
atu2492	*mtbA*	MFS permease	-2.21	3.56E-04
atu4396		O_2-independent ubiquinone biosynthesis accessory factor UbiT	-2.20	2.10E-05
atu0671		conserved hypothetical protein	-2.20	1.90E-06
atu8174		lipid A 4'-phosphatase	-2.18	7.20E-04

Gene locus	Gene name	Description	Fold change	*q* value
atu3588	*alkA*	DNA-3-methyladenine glycosylase II	-2.18	1.30E-04
atu4081		aspartate racemase	-2.17	4.29E-27
atu4604		IS3 family transposase	-2.17	1.51E-19
atu4584		transcriptional regulator, TetR family	-2.08	2.50E-06
atu2364		polar amino acid transport system permease protein	-2.06	2.26E-04
atu1437		conserved hypothetical protein	-2.06	2.84E-04
atu4402		NitT/TauT family transport system permease protein	-2.04	6.67E-04
atu1530	*fixG*	nitrogen fixation protein FixG	-2.03	1.39E-34

TABLE S2 Primers used in this study

Gene-primer name and purpose	Sequence 5′ to 3′
**Gene inactivation**	
triA-BT7876	GAAGGCCGACATTGATGTCG
triA-BT7877	TGAGCTCGGTATAGGACAGC
**Complementation**	
triR-BT7874	ATGGCGAAAGCAAAACTGAC
triR-BT7875	TGGCGATCAGGCCAGTAGAC
**qRT-PCR**	
16S rRNA-BT1421	GAATCTACCCATCTCTGCGG
16S rRNA-BT1422	AAGGCCTTCATCACTCACGC
triA-BT7907	TCCAGACGGATCTGTCGTTC
triA-BT7908	GTCACCTGCGTCTTGAACAG
triR-BT7872	ATTTCGAGACCAAGGAGGCG
triR-BT7873	ACGATCAGGCGGAACAGTTC
atu2198-BT9212	GCGGCTTTTCTTGTCATCGTC
atu2198-BT9213	TGCCGAGACCGATAAATGCTG
tctC-BT8912	GCCGGCATTTCGAGCTATGG
tctC-BT8913	CGACATCAAGGCCGCTTTCC
atu4606-BT9270	GATTGGGGATGGTGCTTGGATC
atu4606-BT9271	CTTCGCCACAACGGCATAAG
nodX-BT9268	ACTCATGCTCCTGTTGCTCC
nodX-BT9269	TGCCCCGTAGGATAAATCGC
atu4608-BT9266	GCTCGACCACACGCATACTAG
atu4608-BT9267	ATCGCTGCTAACCAACGTCAC
atu4609-BT9272	ACCGAGGTCCGAGTGAGTTATG
atu4609-BT9273	AAGTCGGTATCTGCGTGCTC
hslV-BT9990	GGATGCCTTCACGCTGCT
hslV-BT9991	GTGATGGCAAGCGTGGTC
frcC-BT9992	CTGATCGTTCTGGTGCTG
frcC-BT9993	CCGAAAGATCGATACCCG
cysK-BT9302	GAACGCAGAAAGATGCTGGC
cysK-BT9303	TGTCCTGCGGTGAATATCCG
fadD-BT9994	CTGACCTACCGGGAACTC
fadD-BT9995	GAGCGGGTTGACATTGAC
fadB-2-BT9996	CTGTCGATGCTGAAGTCC
fadB-2-BT9997	CATGCAGGTGCCGTTGATG
aglE-BT9404	AGATCTGCTCAAGCTGACGG
aglE-BT9405	TCGTTGGTCGTCCACTTCTG
cysD-2-BT9300	TTTCCTACACCAACCCTCGC
cysD-2-BT9301	GGCGTGCGGAAGGAATAGATTC
cysH-BT9298	AACGGCTTTTACGAGAGCGTC
cysH-BT9299	CTGTTCGATGTCCCAGTCGG
mntH-BT7368	ATTCGGCTACACGCTGCTGG
mntH-BT7369	GCCAAGTTCCAGCGGAATGC
rpoH-BT9998	CGTTATGCCGAGCATGGC
rpoH-BT9999	GAGGCCTTGATCCACCAC
troC-BT3751	TGAAACCGCTCGGCGGTGAG
troC-BT3752	CTGCGCATCCTGCAACATGG
ssuD-BT10k0	GGCTCCGATGATCTGTCG
ssuD-BT10k1	CGGACGAATAGCGACAAG
hspL-BT10k2	GAACAGGCCCAGTCCTATC
hspL-BT10k3	GATACAGGAATTCGCCTTCG
*rbsB-**5**-BT10k4	GAAGTCGTCGTCCAGAGC
*rbsB-**5**-BT10k5	GTGGCGATGACGAGGATG
sitA-BT9306	CCGACACCGCGTGATATTCTC
sitA-BT9307	ATCGCTCACCGTCACATCAG
dctP (*atu5268*)-BT9380	GCAGAACGCATGGCTTTACC
dctP (*atu5268*)-BT9381	TACGCATCTTGAGGCCCTTG
dctQ (*atu5270*)-BT10k6	CTGGCTGCTTCTTGCTGC
dctQ (*atu5270*)-BT10k7	CAGATCGACCTTCACATGC
cspA-4-BT10k8	GGGCTTCGGCTTCATTCAG
cspA-4-BT10k9	GGCGGTCCTGAACGATCTC
dctA-BT10k10	GTCATCTTCCTCACCGTCG
dctA-BT10k11	GGAAGCCGGTGATGGTCTG

**Gene-primer name and purpose**	**Sequence** 5′ to 3′
**qRT-PCR**	
nirV-BT10k12	GCTCCACAGGTCGTTACGG
nirV-BT10k13	GCTGATGCCGGTTGCCGG
norE-BT10k14	CCTTCATCATCGCCTTCGC
norE-BT10k15	CATGGAGCTTCACACCGAC
atu4447-BT9464	GAAGATGCCTGACAACCCGA
atu4447-BT9465	GATGAACGCCATGTTCTCGC
mtlk-BT10k16	GACTTCGCCATCATCGGC
mtlk-BT10k17	GCCCTCGGTAATCGTCATC
cspA-BT10k18	GGGCTTCGGCTTCATTCAG
cspA-BT10k19	CATGTCGCGCTCGAGATCG
**TriR-*Strep*-tagII fusion**	
triR-BT7880	GAGGTTGGTCTCCAATGGCGAAAGCAAAACTGAC
triR-BT7881	GATGACGGTCTCTGCGCTGGCCAGTAGACCGCGCA
